# Phytochemical Composition and Biological Activities of *Scorzonera* Species

**DOI:** 10.3390/ijms22105128

**Published:** 2021-05-12

**Authors:** Karolina Lendzion, Agnieszka Gornowicz, Krzysztof Bielawski, Anna Bielawska

**Affiliations:** 1 Department of Biotechnology, Medical University of Bialystok, Kilinskiego 1, 15-089 Bialystok, Poland; karolina.lendzion@umb.edu.pl (K.L.); aniabiel@umb.edu.pl (A.B.); 2 Department of Synthesis and Technology of Drugs, Medical University of Bialystok, Kilinskiego 1, 15-089 Bialystok, Poland; kbiel@umb.edu.pl

**Keywords:** *Scorzonera*, biological activity, herbal medicine, phytochemical composition, *Asteraceae*

## Abstract

The genus *Scorzonera* comprises nearly 200 species, naturally occurring in Europe, Asia, and northern parts of Africa. Plants belonging to the *Scorzonera* genus have been a significant part of folk medicine in Asia, especially China, Mongolia, and Turkey for centuries. Therefore, they have become the subject of research regarding their phytochemical composition and biological activity. The aim of this review is to present and assess the phytochemical composition, and bioactive potential of species within the genus *Scorzonera*. Studies have shown the presence of many bioactive compounds like triterpenoids, sesquiterpenoids, flavonoids, or caffeic acid and quinic acid derivatives in extracts obtained from aerial and subaerial parts of the plants. The antioxidant and cytotoxic properties have been evaluated, together with the mechanism of anti-inflammatory, analgesic, and hepatoprotective activity. *Scorzonera* species have also been investigated for their activity against several bacteria and fungi strains. Despite mild cytotoxicity against cancer cell lines in vitro, the bioactive properties in wound healing therapy and the treatment of microbial infections might, in perspective, be the starting point for the research on *Scorzonera* species as active agents in medical products designed for miscellaneous skin conditions.

## 1. Introduction

*Scorzonera* L. is a genus in the *Cichorieae* tribe of the *Asteraceae* family. It is spread mostly in central and southern parts of Europe, Eurasia, and Africa in arid areas [1,2]. Numerous species are endemic to Anatolia (Turkey) [1,3,4,5,6,7,8,9,10,11], Mongolia [12,13,14,15], and China [16,17,18] The genus comprises approximately 180–190 species [19], including *S. hispanica,* whose roots are a valued vegetable, with the taste similar to asparagus [20], and *S. tau-saghyz* (a species of interest in terms of obtaining natural rubber) [21]. Several *Scorzonera* species are a source of feed for farming animals in arid regions [15]. Typically, plants within the *Scorzonera* genus are perennial herbs characterized by the presence of a caudex or tuber. Biennial plants or dwarf subshrubs are rare [19]. Plants within the genus *Scorzonera* are reported to contain flavonoids [12,13,22,23,24], phenolic acid derivatives [8,13,25,26], triterpenoids [18,23,27,28,29,30,31], sesquiterpenoids [14,17,20,32,33,34], dihydroisocoumarins [7,35,36,37], and other bioactive compounds. *Scorzonera* species have been commonly used as medicinal plants in European and Asian herbal therapy for ages. In Turkey, they are known as hemostatic agents, as well as, when used externally, as plasters in the process of wound healing [38]. The plants are also present in folk medicine as a remedy for hypertension, atherosclerosis, or kidney dysfunction [39].

This review aims to present the phytochemical composition of *Scorzonera* species, including the compounds characteristic for the genus, as well as novel compounds, which have not been previously isolated from *Asteraceae*. Extraction methods have been briefly summarized as well. A summary of available data regarding the use of *Scorzonera* in folk medicine has also been included. Phytochemical composition and ethnopharmacological reports lead to the third part of this paper, the assessment of biological activities of natural products (extracts, fractions, and pure compounds) obtained from species within the genus *Scorzonera.* To our best knowledge, this is the first comprehensive review of the current findings in the field of phytochemistry and bioactivity of *Scorzonera* species. The assessment of biological activity in vitro and in vivo is the first step in the development of new plant-derived products and those play a substantial role in healthcare [40]. Some novel natural medicines are under clinical trials [41], others have been approved in therapy [42,43]. In this paper, a summary of the results of in vitro and in vivo studies has been made, as well as an attempt to evaluate their significance and therapeutic potential.

The search strategy for this review involved browsing results for terms ‘*Scorzonera*’ and ‘biological activity’ or ‘bioactive’, ’*Scorzonera*’ and ‘phytochemistry’ ‘phytochemical composition’ in the following databases: Reaxys, PubMed, and ScienceDirect. The search was limited to the years 2000–2021, with three studies published before the year 2000 included in this review [44,45,46].

## 2. *Scorzonera* in Traditional Medicine

Genera within the family *Asteraceae* have been present in folk medicine across Europe, Asia, and northern Africa. That includes species within the *Scorzonera* genus, which are a significant part of Turkish traditional medicine in the therapy of arteriosclerosis, kidney disorders, wounds, rheumatism, but also as antidiabetic, antihypertensive, and antinociceptive medications [5]. The leaves of *S. latifolia* (Fisch and Mey.) DC., applied topically, act as plaster and prevent nausea. Turkish folk medicine uses latex obtained from *S. latifolia* to treat infertility and as an anthelmintic and pain-relieving medication [3,6,38,47]. Roots of *S. tomentosa* L. are believed to have hemostatic properties when ingested [38]. Aerial parts of *S. laciniata* L. are known as antipyretic, antipyogenic, antiatherosclerotic, antidiabetic, antirheumatic, and blood pressure-lowering agents in folk therapy [39]. Turkish folk medicine uses *S. phaeopappa* Boiss., *S. sosnowskyii* Lipsch., and *S. mirabilis* Lipsch. for headaches. *S. mollis* Biela is used as a diuretic and against kidney stones [48]. In Algerian traditional medicine, *S. undulata* ssp. *deliciosa* is a part of the treatment of snake bites [49]. Mongolian folk remedies for various ailments include *Scorzonera* species as well. There are reports for the use of *S. pseudodivaricata* Lipsch. as antipyretic in viral and bacterial infections, anti-diarrheal and diuretic agents, as well as for the treatment of lung edema and diseases caused by parasite infections. Aerial and subaerial parts of *S. divaricata* Turcz. are used to treat ulcers and stomach tumors. [14]. Leaves and shoots of *S. divaricata* are also present in the folk medicine of India in the therapy of jaundice [50]. Traditional Chinese medicine uses *S. mongolica* Maxim. root to reduce fever and treat carbuncle mastitis, as well as an antineoplastic agent [31]. Roots of *Scorzonera hispanica* L., currently cultivated and eaten as a vegetable, were formerly used in European folk medicine as a mucolytic agent in pulmonary diseases, appetite stimulator, and to defeat a cold. [14,32]. Tibetan folk medicine has used *S. austriaca* Willd. for the treatment of carbuncle, inflammation, and fever [17,28]. *Scorzonera radiata* Fisch. is a Mongolian traditional remedy for bacterial and viral infection-induced fever, poisonous ulcers, and as a lactation-inducing and diuretic agent [15]. In Libya, *Scorzonera resedifolia* L. is known as a folk medication for liver pain [51].

## 3. Phytochemical Composition of *Scorzonera* Species

Species within the *Scorzonera* genus are a source of flavonoid aglycones and glycosides, phenolic acids and their derivatives, lignans, triterpenoids, sesquiterpenoids, dihydroisocoumarins, bibenzyl derivatives, as well other compounds [4,10,14,27,35,36,52,53,54,55].

### 3.1. Scorzonera acuminata Boiss.

The samples of *Scorzonera acuminata* aerial parts and roots were collected in the northern part of Anatolia, Turkey. In the study by Süntar et al. [9], plant samples were extracted with 20% aqueous methanol. The study by Bahadır-Acıkara et al. [27] includes a phytochemical analysis of *n*-hexane extracts of *S. acuminata* aerial parts and roots.

A 20% aqueous methanol extract of the aerial parts of *S. acuminata* was reported to contain chlorogenic acid, rutin, and cyranoside [9]. Another study reports the presence of α-amyrin, lupeol, and lupeol acetate in the *n*-hexane extract [27].

In the aqueous methanol extract from the roots, chlorogenic acid and trace amounts of rutin were found [9]. An *n*-hexane extract contained lupeol, lupeol acetate, and α-amyrin [27].

### 3.2. Scorzonera aristata Rameond ex DC.

Samples of *S. aristata* were collected in Northern Italy [26,55,56]. The leaves from the specimen in the study by Jehle et al. [56] were extracted with methanol and subsequently with a mixture of methanol, acetone and water (3/1/1, *v*/*v*/*v*), the roots were first treated with the mixture of methanol, acetone, and water (3/1/1, *v*/*v*/*v*), then were extracted with methanol. The subaerial parts of the samples in the study by Zidorn et al. [55] were mixed with a stock solution and sonicated with methanol, then HPLC was performed. A study by Granica and colleagues [26] involved extraction of aerial parts of the species with 50% aqueous methanol and the HPLC analysis of obtained extracts.

From the aerial part extracts of the plant, flavonoids (rutin, isoorientin, and quercetin 3-*O*-glucoside) and caffeic acid derivatives (chlorogenic acid, 3,5-dicaffeoylquinic acid, 4,5-dicaffeoylquinic acid) were isolated [56]. In the 50% aqueous methanol extract, the presence of chlorogenic acid, 4-*O*-caffeoylquinic acid, 1,5-*O*-dicaffeoylquinic acid, and rutin, as well as apigenin derivative and luteolin derivative [26].

From subaerial parts, 3,5-dicaffeoylquinic acid and caffeic acid methyl ester were isolated, along with the following triterpenes: lupeol, magnificol, and 3α-hydroxyolean-5-ene [56]. The presence of chlorogenic acid and 3,5-dicaffeoylquinic acid was previously reported in the study by Zidorn et al. [55].

### 3.3. Scorzonera aucheriana DC.

Samples of *S. aucheriana* were collected in central Turkey and aerial parts were extracted with methanol at room temperature [7,29].

Investigation on aerial parts of the plant led to the isolation of dihydroisocoumarins and dihydroisocoumarin derivatives (scorzopygmaecoside, scorzocreticoside II, iso-scorzopygmaecoside, scorzoaucherioside I and II), quinic acid derivatives (3,5-*O*-dicaffeoyl-*epi*-quinic acid and 3,5-*O*-dicaffeoylquinic acid), and 3,4-dihydroxyphenyl caffeate [7]. In another study, chlorogenic acid derivatives (methyl 1-(2-methylcyclopropyl-1-carbonyloxy)chlorogenate and 3,4-bis[(3′,4-dioxo-1′,3′,5′,6′-tetrahydrospiro[cyclohexa-2,5-diene-1,4′-cyclopenta[*c*]-furan]-1′-yl)]chlorogenic acid), triterpenoids (taraxasterol, taraxasterol acetate, taraxasterol oleate, lupeol, lupeol acetate and ptiloepoxide) and β-sitosterol were isolated from the methanol extract of *S. aucheriana* aerial parts [29].

### 3.4. Scorzonera austriaca Willd.

Samples of *S. austriaca* were collected in the northeast [24] and central [17,33] parts of China.

Herbs of *S. austriaca* were extracted with 70% aqueous ethanol. The extract was reported to contain the following flavonoid glycosides and flavonoid glycoside derivatives: 5,7,4′-trihydroxyflavone 6-*C*-(2″-*O*-β-D-glucopyranosyl β-D-glucopyranoside), 5,7,3′,4′-tetrahydroxyflavone 6-*C*-(2″-*O*-β-D-glucopyranosyl β-D-glucopyranoside), quercetin 3-*O*-rutinoside, 5,7,4′-trihydroxyflavone 6-*C*-β-D-glucopyranoside, 3′-methoxy-5,7,4′-trihydroxyflavone 6-*C*-β-D-glucopyranoside, 5,7,4′-trihydroxyflavone 8-*C*-(6″-*O*-*trans*-caffeoyl β-D-glucopyranoside and 5,7,3′,4′-tetrahydroxyflavone 8-*C*-(6″-*O*-*trans*-caffeoyl β-D-glucopyranoside [24].

Roots of the plant samples collected in central China were extracted with acetone and guaianolides (biguaiascorzolide A and biguaiascorzolide B) were isolated [17]. Before that discovery, in 2004, Li et al. [16] isolated a sesquiterpene lactone (3β,11α-dihydroxy-4β-methyl-guaia-10 (14)-en-12, 6α-olide) from the acetone extract of *S. austriaca* roots. Other identified in the root acetone extract sesquiterpenoids were: scorzoaustriacoside, scorzoaustriacin, scorzoaustriacin 3-*O*-β-D-glucoside, 4-*epi*-dihydroestafiatol, 14-isovaleroxyscorzoaustricin, 14-isovaleroxyscorzoaustricin sulfate, zaluzanin C, glucozaluzanic C, dehydrocostus lactone, 11β,13-dihydrozaluzanin and diacetoxyisolippidiol [33]. In the study by Wu et al. from 2011 [28], the following compounds were isolated from the acetone root extract: oleanane-type triterpenes (3β-acetoxyglutin-5(10)-en-6-oxo, glutinol, β-amyrin-3-(3′-methylbutanonate), β-amyrin 3-acetyl, 3β-acetyl-11α,12α-oxidotaraxerol), ursane-type triterpenes (α-amyrin 3-acetyl, α-amyrin 3-acetyl-11-oxo, D-friedours-14-en-3β-acetyl-11α,12α-epoxy, taraxasterol, and ψ-taraxasteryl 3(3′-methyl-butanonate)), lupeol, (23*Z*)-cycloart-23-ene-3β, 25-dihydroxy, 9β,19-cyclolanostane-24-en-3-*oxo*, and steroids (β-sitosterol, β-stigmasterol, stigmast-4-en-3-one, stigmast-3β,5α,6β-trihydroxy, and β-sitosterol 3-β-D-glucoside).

### 3.5. Scorzonera baetica (Boiss.) Boiss.

Aerial parts of plant samples, collected in Spain, were extracted with 50% aqueous methanol at room temperature and the extracts were analyzed using HPLC.

The aerial part 50% methanol extract was reported to contain caffeoylquinic acid derivatives (3-*O*-caffeoylquinic acid, 4-*O*-caffeoylquinic acid, chlorogenic acid, 1,5-*O*-dicaffeoylquinic acid, 3,5-*O*-dicaffeoylquinic acid, 4,5-*O*-dicaffeoylquinic acid), flavonoid glycosides (orientin, isoorientin, vitexin, isovitexin, cyranoside), and flavonoid diglycosides [26].

### 3.6. Scorzonera cana (C.A. Meyer) Hoffm. var. alpina (Boiss.) Chamb.

Plant samples were collected in the north-central part of Turkey and extracted with 20% aqueous methanol at room temperature.

In the extract of the aerial parts, rutin and notable amounts of chlorogenic acid were reported present.

The presence of chlorogenic acid was detected in the root extract [9].

### 3.7. Scorzonera cana (C.A. Meyer) Hoffm. var. jacquiniana (W. Koch) Chamb.

Plant samples were collected in central Turkey [9,27]. Aerial and subaerial parts were separated and extracted with *n*-hexane [27] and 20% aqueous methanol [9].

Triterpenoids present in the *n*-hexane extract of the aerial parts were taraxasteryl acetate, lupeol, lupeol acetate, and α-amyrin [27]. Compounds present in the aqueous methanol extract were: chlorogenic acid, rutin, hyperoside, luteolin 7-glucoside, and trace amounts of apigenin [9].

In the root aqueous methanol extracts, chlorogenic acid was found. [9]. There were also reports on the presence of α-amyrin. taraxasteryl acetate, lupeol, and lupeol acetate in the *n*-hexane extract [27].

### 3.8. Scorzonera cinerea Boiss.

Samples of *S. cinerea* collected in central Turkey were extracted with *n*-hexane at room temperature.

In the extract of the aerial parts of *S. cinerea,* the following triterpenoids were detected: lupeol, lupeol acetate, taraxasteryl acetate, 3β-hydroxy-fern-7-en-6-one-acetate, α-amyrin, and olean-12-en-11-one-3-acetyl.

In the root extract of the plant, taraxasteryl acetate, lupeol, lupeol acetate, and α-amyrin were detected [27].

### 3.9. Scorzonera cretica Willd.

Samples of *S. cretica* were collected on Crete, Greece. The whole plant was subjected to extraction with dichloromethane and subsequently with methanol.

From the dichloromethane extract, dihydroisocoumarin (scorzocreticin), dihydroisocoumarins glycosides (scorzocreticoside I, scorzocreticoside II), and 3-*O*-β-D-glucopyranosylsitosterol were isolated. Triterpenoids (lupeol, lupeol acetate, lupenone, germanicol, germanicol acetate, germanicone, taraxasterol, taraxasterol acetate, oleanol, oleanol acetate) were isolated from the methanol extract of the plant [37].

### 3.10. Scorzonera crispatula Boiss.

Plant samples (aerial parts) were collected in England [44] and Spain. Samples from Spain were extracted at room temperature with a mixture of methanol and water (1:1, *v*/*v*) and subjected to HPLC [26]. Samples collected in England were extracted with ethanol [44].

In the *S. crispatula* aerial part hydromethanolic extract, the presence of caffeic acid derivatives (3-*O*-caffeoylquinic acid, 4-*O*-caffeoylquinic acid, chlorogenic acid, 1,5-*O*-dicaffeoylquinic acid. 3,5-*O*-dicaffeoylquinic acid and 4,5-*O*-dicaffeoylquinic acid), flavonoid aglycones (quercetin and luteolin) flavonoid C-glycosides (isoorientin and isovitexin), and several flavonoid diglycosides was reported [26]. In the ethanol extract of *S. crispatula*, luteolin and quercetin were detected.

### 3.11. Scorzonera divaricata Turcz.

Samples of *S. divaricata* were collected in Mongolia [14] and central China [34,57,58,59]. Aerial parts were extracted with methanol at room temperature [14,34], then with methanol at 65 °C [34]. Subaerial parts were subjected to extraction with 95% aqueous ethanol [57,58,59].

From the aerial part methanol extract, feruloylpodospermic acid A and feruloylpodospermic acid B were isolated and the presence of known compounds (scopoletin, chlorogenic acid, isovitexin 4′-O-glucoside, isovitexin 2′-O-xyloside, kaempferol 3-O-rutinoside, apigenin) was detected [14]. The aerial part methanol extract was used to isolate the novel compounds sulfoscorzonin D and sulfoscorzonin E. Apart from sulfoscorzonins, the following compounds were isolated: benzoic acid derivatives (methyl-3,4-dihydroxybenzoate, *m*-hydroxybenzoic acid), coumarin derivatives (scopoletin, 7-hydroxycoumarin), flavonoid aglycones (diosmetin, luteolin, tricin, 7,3′,4′-trihydroxyflavonol, 5,7-dihydroxy-8-methoxyflavone, 5,7-dihydroxy-6-methoxyflavone), phenolic acid derivatives (*trans*-caffeic acid, *trans*-*p*-hydroxycoumaric acid, 4-hydroxy-3-methoxyphenyl ferulate), sesquiterpenoids (glucozaluzanin C, 1β,4α-dihydroxy-5α,6β,7α,11β*H*-eudermn-12,6-olide), steroids ((22*E*)-5α,8α-epidioxyergosta-6,22-dien-3β-ol, ergosta-3β,5α,6β-trialcohol, stigma-5-en-3-*O*-β-glucoside), triterpenoids (oleanolic acid and lup-20(29)-ene-3β,28-diol), sacrolide A, and vomifoliol [34].

From the subaerial part ethanolic extract, the following compounds were isolated: phenolic acids and their derivatives ((–)–1,4-di-*O*-feruloyl-3-*O*-dihydrocaffeoylquinic acid, (–)–1-O-feruloyl-4-O-dihydrocaffeoylquinic acid (–)–3,5-di-O-feruloylquinic acid, (–)–1-*O*-feruloyl-3-*O*-dihydrocaffeoylquinic acid, (–)–1-*O*-feruloyl-5-*O*-dihydrocaffeoylquinic acid, 3-*O*-feruloylquinate, butyl 3-*O*-feruloylquinate, caffeic acid, dihydrocaffeic acid and its methyl, ethyl and n-butyl esters), triterpenoids (scorzodivaricin A, scorzodivaricin B and scorzodivaricin C, scorzodivaricin D, 23(Z)-3β-acetoxy-25-hydroxy-tirucalla-7,23-diene, 23(*Z*)-3β,25-dihydroxy-tirucalla-7,23-diene, 23(*Z*)-3β,25-dihydroxy-tirucalla-7,23-diene, 20(*R*)-3β-acetoxy21-hydroxy-24(31)-methylene-dammarane and oleanolic acid), sesquiterpenoids (sulfoscorzonin A, sulfoscorzonin B, sulfoscorzonin C, and 10(*Z*)-1-oxo-bisabola-2,10-dien-13-al), steroids (5α,8α-epidioxy-(22*E*,24*R*)-ergosta-6,22-dien-3β-ol, stigmast-4-en-6β-ol-3-one 6β-hydroxystigmastan-4-en-3-one, 3β-hydroxystigmast-5-en-7-one, 5,6α-epoxy-5α-stigmastan-3β-ol, 7β-hydroxysitosterol, 7α-hydroxysitosterol, β-sitosterol, and β-daucosterol), benzene derivatives (vanillin, vanillic acid 4-*O*-β-D-glucoside, vanillic acid 1-*O*-β-D-glucopyranosyl ester, tachioside, syringic acid ethyl ester, and 3,4-dimenthoxy-3′-hydroxy propiophenone), fatty acids (pinellic acid, linoleic acid, and palmitic acid), coumarin derivatives (scopolin and scopoletin), and a lignan (pinoresinol) [57,58,59].

### 3.12. Scorzonera eriophora DC.

Samples of *S. eriophora* aerial and subaerial parts were collected in Turkey and extracted at room temperature with 20% aqueous methanol [9] and *n*-hexane [27].

Chlorogenic acid was detected in aqueous methanol extracts of both aerial parts and roots. The aerial part extract was also reported to contain luteolin and luteolin 7-glycoside [9]. The *n*-hexane extracts of aerial and subaerial parts both contained taraxasteryl acetate, lupeol, lupeol acetate Additionally, 3β-hydroxy-fern-7-en-6-one-acetate was reported to be present in the *n*-hexane root extract [27].

### 3.13. Scorzonera graminifolia L.

Aerial parts of the plant, collected in England, were extracted with ethanol and reported to contain quercetin and luteolin [44].

### 3.14. Scorzonera hieraciifolia Hayek

Samples of the plant were collected in central Turkey. Aerial and subaerial parts were separated, and aerial parts were extracted with ethanol at room temperature and fractioned. Then isolation of compounds was performed.

From the subaerial part ethanol extract, the following compounds were isolated: quinic acid derivatives (5-*O*-feruloyl quinic acid methyl ester, 1,5-di-*O*-feruloylquinic acid, chlorogenic acid methyl ester, 3-*O*-caffeoylquinic acid methyl ester, 1,3-di-*O*-caffeoylquinic acid methyl ester, 3,5-di-*O*-caffeoylquinic acid methyl ester, and 4,5-di-*O*-caffeoylquinic acid methyl ester), caffeic acid, and 3-(4′-hydroxyphenyl)-2-propenoic acid (4″-carboxyl)-phenyl ester [8].

### 3.15. Scorzonera hirsuta L.

Samples of *S. hirsuta* aerial parts, collected from the University of Reading (Reading, UK), were extracted with alcohol and the extract was reported to contain flavonoid aglycones: kaempferol, luteolin, and quercetin [44].

### 3.16. Scorzonera hispanica L.

In the studies by Granica et al. [20,26], plant samples were collected in Germany, subaerial parts for the quantitative analyses were purchased in Austria and Warsaw. In the study by Zidorn et al. [32], plant samples from Belgium were used. In the study by Petkova [60], the plant was harvested in Bulgaria.

For the isolation and identification of major constituents of *S. hispanica* subaerial parts, maceration with ethyl acetate was carried out. The phenolic compounds in aerial and subaerial parts were quantified using a modification of a method described by Zidorn et al. [61] with a mixture of methanol/acetone/water (3:1:1) [20]. For the elucidation of inulin content, the roots were extracted with water via microwave-assisted extraction [60].

In the aerial part aqueous methanol extract, the following compounds were detected: flavonoid glycosides (isoorientin, hyperoside, isoquercitrin, miquelianin), luteolin di-*C*-glycoside (*C*-hexoside, *C*-pentoside), quercetin, caffeic acid, and caffeic acid derivatives (chlorogenic acid, 4-*O*-caffeoylquinic acid 1,5-*O*-dicaffeoylquinic acid, 3,5-*O*-dicaffeoylquinic acid, 4,5-*O*-dicaffeoylquinic acid, 3-*O*-caffeoylquinic acid, 4-*O*-caffeoylquinic acid, 1,5-*O*-dicaffeoylquinic acid, 3,5-*O*-dicafeoylquinic acid, 4,5-*O*-dicaffeoylquinic acid) [20,26].

From the methanol extract of *S. hispanica* subaerial parts, plugitone, ixerioside D, and 3-*O*-angeloyl-11β,13-dihydrodesacylcynaropicrin-8β-D-glucoside were isolated [32].

Subaerial part ethyl acetate extract was reported to contain a lignan ((‒)-syringaresinol), octadecadienoic acids (linoleic acid, 9-hydroxyocta-(10*E*,12*E*)-decadienoic acid, 8-13-*oxo*-(9*Z*,11*E*)-octadecadienoic acid, 9-*oxo*-(10*E*,12*Z*)-octadecadienoic acid, 13-*oxo*-(9*E*,11*E*)-octadecadienoic acid, and 9-*oxo*-(10*E*,12*E*)-octadecadienoic acid), and sesquiterpenoids (1-*oxo*-bisabola-(2,10*E*)-diene-12-carboxylic acid, 1-*oxo*-bisabola-(2,10*E*)-diene-12-ol, plitostemonol, puliglutone, 1-*oxo*-bisabola-(2,10*E*)-diene-12-carboxylic acid methyl ester, 2,9-epoxycurcumen-12-al, and ixerisoside D) [20].

Caffeic acid and caffeic acid derivatives (chlorogenic acid, 4-*O*-caffeoylquinic acid, 1,5-*O*-dicaffeoylquinic acid, 3,5-*O*-dicaffeoylquinic acid, and 4,5-*O*-dicaffeoylquinic acid) were identified in subaerial part samples as well [20]. Notable amounts of inulin (over 20% of dry plant material) were identified in the roots of *S. hispanica* [60].

### 3.17. Scorzonera humilis L.

Samples of *S. humilis* were collected in Austria and subaerial parts of the plant were extracted with methanol [53,54,55].

From the methanol extract, tyrolobibenzyls were isolated via column chromatography [53,54,55]. Tyrolobibenzyls A, B, and C were isolated and identified in the study from 2000, together with a lignin—pinoresinol-1-yl β-D-glucopyranoside [54]. In the study from 2002, the structure of tyrolobibenzyl D was elucidated [53]. A year later, the structure of two novel tyrolobibenzyls (E and F) was identified and the presence of chlorogenic acid and 3,5-dicaffeoylquinic acid was detected [55].

### 3.18. Scorzonera incisa DC.

Samples of the plant were collected in Turkey and aerial and subaerial parts were extracted separately at room temperature with 20% aqueous methanol [5] and *n*-hexane [27].

The aerial part *n*-hexane extract was reported to contain triterpenes: lupeol, lupeol acetate, α-amyrin, and taraxasteryl acetate [27]. Additionally, the presence of rutin, cyranoside, and chlorogenic acid was detected in the aqueous methanol extract [5].

In the root extracts, the presence of chlorogenic acid was detected in the aqueous methanol extract [5]. Triterpenoids (lupeol and lupeol acetate, taraxasteryl acetate, and olean-12-en-11-one-3-acetyl) were reported present in the *n*-hexane root extract [5,27].

### 3.19. Scorzonera judaica Eig.

The roots of the plant were collected in Jordan and subsequently extracted with *n*-hexane, chloroform, a mixture of chloroform and methanol (9:1), and methanol. Then, the isolation of compounds was carried out.

From the chloroform root extract, 4α-hydroxypinoresinol, hydrangenol, and scorzotomentosin were isolated.

The CHCl_3_:MeOH (9:1) extract was reported to contain 3*S*-hydrangenol 4′-*O*-α-L-rhamnopyranosyl-(1→3)-β-D-glucopyranoside, hydrangenol 4′-*O*-β-D-apiofuranosyl-(1→6)-β-D-glucopyranoside, 2-hydroxy-6-[2-(4-hydroxyphenyl)-2-*oxo*-ethyl]benzoic acid, *E*-3-(3,4-dihydroxybenzylidene)-5-(3,4-dihydroxyphenyl)dihydrofuran-2-one, *Z*-3-(3,4-dihydroxybenzylidene)-5-(3,4-dihydroxyphenyl)-2(3*H*)-furanone, 4-[β-D-glucopyranosyl)hydroxy]-pinoresinol, hydrangenol 8-*O*-β-D-glucopyranoside, hydramacrophyllol A, hydramacrophyllol B, 4α-hydroxypinoresinol, hydrangenol 4′-*O*-β-D-glucopyranoside, thunberginol F, and hydrangenol.

From the methanol extract, the following compounds were isolated: 3*S*-hydrangenol 4′-*O*-α-L-rhamnopyranosyl-(1→3)-β-D-glucopyranoside, hydrangenol 4′-*O*-β-D-apiofuranosyl-(1→6)-β-D-glucopyranoside, thunberginol F 7-*O*-β-D-glucopyranoside, 2-hydroxy-6-[2-(4-hydroxyphenyl)-2-*oxo*-ethyl]benzoic acid, 2-hydroxy-6-[2-(3,4-dihydroxyphenyl)-2-*oxo*-ethyl]benzoic acid, 2-hydroxy-6-[2-(3,4-dihydroxyphenyl-5-methoxy)-2-oxoethyl]benzoic acid, hydrangeic acid 4′-*O*-β-D-glucopyranoside, 4-[β-D-glucopyranosyl)hydroxy]-pinoresinol, hydrangenol 8-*O*- β-D-glucopyranoside, and hydrangenol 4′-*O*-β-D-glucopyranoside [25].

### 3.20. Scorzonera laciniata L. ssp. laciniata

Plant samples were collected in the north-western [9,27] and eastern parts of Turkey. [22]. Aerial and subaerial parts were separated and extracted with 20% aqueous methanol [9] and *n*-hexane [27]. In the study by Erden et al. [22], the extraction was carried out using methanol, a mixture of hexane and isopropyl alcohol (3:2, *v*/*v*), water, and a mixture of HNO_3_:H_2_SO_4_:H_2_O_2_ (10:1:1, *v*/*v*/*v*).

In the aerial part 20% aqueous methanol extract, chlorogenic acid and luteolin 7-glucoside, as well as trace amounts of rutin were reported [9]. Myricetin, kaempferol, and trace amounts of morin and quercetin were present in the methanol extract. Phytosterols (ergosterol, stigmasterol, and β-sitosterol) and vitamins D and K were identified in the hexane/isopropyl alcohol (3:2, *v*/*v*) extract and notable amounts of potassium were identified in the extract obtained with the mixture of HNO_3_:H_2_SO_4_:H_2_O_2_ [22]. The presence of lupeol, lupeol acetate, taraxasteryl acetate, and α-amyrin in the *n*-hexane aerial part extract has been reported as well [27].

Root aqueous methanol extract contains chlorogenic acid [9]. In the *n*-hexane extract of the roots, triterpenoids (taraxasteryl acetate, lupeol, lupeol acetate, and α-amyrin) were detected [27].

### 3.21. Scorzonera latifolia (Fisch. and Mey.) DC.

Samples of *S. latifolia* were collected in eastern [5,27] and central-eastern Turkey [10,22]. Aerial and subaerial parts were separated and extracted with 20% aqueous methanol [5], and *n*-hexane [27]. The study by Erden et al. [22] reports extraction with methanol, a mixture of hexane and isopropyl alcohol (3:2, *v*/*v*), water, and a mixture of HNO_3_:H_2_SO_4_:H_2_O_2_ (10:1:1, *v*/*v*/*v*).

Aerial part methanol extract of *S. latifolia* was reported to contain flavonoid aglycones: myricetin, quercetin, kaempferol, and morin. Ergosterol, stigmasterol, and β-sitosterol were also reported present in the hexane:isopropyl alcohol (3:2, *v*/*v*) extract from the aerial parts of the plant, together with retinol and vitamins D, E, and K [22]. Triterpenoids identified in aerial part *n*-hexane extract were: taraxasteryl acetate, 3β-hydroxy-fern-7-en-6-one-acetate, lupeol, lupeol acetate, and α-amyrin [27]. The aqueous methanol extract from the aerial parts was reported to contain chlorogenic acid, hyperoside, and luteolin 7-glucoside [5].

In the roots, the following triterpenoids are reported to be present in the *n*-hexane extract: taraxasteryl acetate, lupeol, lupeol acetate, β-hydroxy-fern-7-en-6-one-acetate, and olean-12-en-11-one-3-acetyl [27]. Moreover, chlorogenic acid was detected in the 20% aqueous methanol extract [5].

### 3.22. Scorzonera mirabilis Lipsch.

Samples of the plant were collected in the city of Van, eastern Turkey, aerial parts and roots were separated in extracted at room temperature with *n*-hexane.

Both aerial and subaerial part *n*-hexane extracts of *S. mirabilis* contain taraxasteryl acetate, lupeol, and lupeol acetate [27].

### 3.23. Scorzonera mollis Bieb. ssp. szowitsii (DC.) Chamb.

Plants samples were collected in north-central Turkey [5,27]. Aerial and subaerial parts were separated and extracted at room temperature using *n*-hexane [27] and 20% aqueous methanol [5].

In the aqueous methanol extract of aerial parts, chlorogenic acid, rutin, hyperoside, and cyranoside were detected [5]. The presence of taraxasteryl acetate, lupeol, lupeol acetate, and α-amyrin was reported in *n*-hexane extracts from both aerial and subaerial parts of the plant [27].

Aqueous methanol root extract was reported to contain chlorogenic acid [5].

### 3.24. Scorzonera papposa DC.

Samples were collected in Jordan. Aerial parts and roots were separated, dried, and sequentially macerated with *n*-hexane, chloroform, a mixture of chloroform and methanol (9:1), and methanol.

From the aerial part methanol extract, the following compounds were isolated: (6-*trans*-*p*-coumaroyl)-3-*O*-β-D-glucopyranosyl-2-deoxy-D-riburonic acid, a mixture of (6-*cis*-*p*-coumaroyl)-3-*O*-β-D-glucopyranosyl-2-deoxy-D-riburonic acid and (6-*cis*-*p*-coumaroyl)-3-*O*-β-D-glucopyranosyl-2-deoxy-D-ribono-*c*-lactone, (6-*trans*-*p*-coumaroyl)-3-*O*-β-D-glucopyranosyl-2-deoxy-D-riburonic acid methyl ester, (6-*trans*-*p*-coumaroyl)-3-*O*-β-D-glucopyranosyl-(5-acetyl)-2-deoxy-D-riburonic acid, isoorientin, orientin, isoschaftoside, and swertiajaponin. From the CHCl_3_:MeOH extract, (6-*trans*-*p*-coumaroyl)-3-*O*-β-D-glucopyranosyl-2-deoxy-D-riburonic acid, a mixture of (6-*cis*-*p*-coumaroyl)-3-*O*-β-D-glucopyranosyl-2-deoxy-D-riburonic acid and (6-*cis*-*p*-coumaroyl)-3-*O*-β-D-glucopyranosyl-2-deoxy-D-ribono-c-lactone, and (6-*trans*-*p*-coumaroyl)-3-*O*-β-D-glucopyranosyl-(5-acetyl)-2-deoxy-D-riburonic acid were isolated.

The root methanol extract was reported to contain thunberginol G [62].

### 3.25. Scorzonera parviflora Jacq.

Aerial and subaerial parts of the plant, collected in central Turkey, were separated and extracted at room temperature using *n*-hexane [27] and 20% aqueous methanol [5].

Aerial part *n*-hexane extract of *S. parviflora* was reported to contain the following triterpenoids: taraxasteryl acetate, lupeol, and lupeol acetate [27]. The aqueous methanol extract contained chlorogenic acid, hyperoside, and cynaroside [5].

In the roots, chlorogenic acid was detected in the aqueous methanol extract [5]. Taraxasteryl acetate, lupeol, and lupeol acetate were the main components of the *n*-hexane extract [27].

### 3.26. Scorzonera pseudodivaricata Lipsch.

Samples for the analysis were collected in Mongolia. Aerial and subaerial parts of the plant were separated, then aerial parts were macerated with methanol at room temperature.

Aerial part extract of the plant is reported to contain isochlorogenic acid A, cynaroside, isovitexin 2″-*O*-xyloside, luteolin, luteolin 5-*O*-glucoside, platyphylloside, scopoletin, scorzoneric acid and scorzonerin [14].

### 3.27. Scorzonera pusilla Pall.

In the aerial parts, collected in Reading (England), the presence of quercetin and luteolin was reported [44].

### 3.28. Scorzonera pygmaea Sibth. and Sm.

Plant samples (subaerial parts) were collected in Turkey. The dried and powdered subaerial parts were macerated in ethanol.

From the subaerial part methanol extract, the following compounds were isolated: 3,5-dicaffeoylquinic acid, chlorogenic acid, chlorogenic acid methyl ester, cudrabibenzyl A, scorzocreticoside I scorzocreticoside II, scorzonerol, scorzopygmaecoside, and thunberginol C [35].

### 3.29. Scorzonera radiata Fisch.

Samples of *S. radiata* aerial parts were collected in Mongolia and macerated with methanol at room temperature [12,13,15].

From the aerial part methanol extract of *S. radiata,* scorzodihydrostibenes A-E were isolated [15]. Apart from that, the presence of 3,5-dicaffeoyl-*epi*-quinic acid, 3,5-dicaffeoylquinic acid, 4,5-dicaffeoylquinic acid, 5-*p*-coumaroylquinic acid (*cis* and *trans*), chlorogenic acid, isoorientin, kaempferol 3-*O*-rutinoside, macroantoin F, macroantoin G, quinic acid, rutin, and violanthin was detected [12], Moreover, scorzonerin A, scorzonerin B, and 4,5-dicaffeoyl-*epi*-quinic acid were isolated [13].

### 3.30. Scorzonera suberosa C. Koch ssp. suberosa

Samples of the plant were collected in the central part of Turkey. Aerial and subaerial parts were separated and extracted with *n*-hexane at room temperature [27]. In the study by Erden et al. [22], the solvents used for extraction were methanol, a mixture of hexane and isopropyl alcohol (3:2, *v*/*v*), water, and a mixture of HNO_3_:H_2_SO_4_:H_2_O_2_ (10:1:1, *v*/*v*/*v*).

Aerial parts and roots are reported to contain taraxasteryl acetate, lupeol, and lupeol acetate [27]. Myricetin, morin, and quercetin were found in the methanol extract, vitamins D, E, and K, retinol, and phytosterols: β-sitosterol, ergosterol, and stigmasterol were detected in the hexane/isopropyl alcohol extract. Sodium and potassium were also reported present (extraction using the mixture of HNO_3_:H_2_SO_4_:H_2_O_2_) [22].

### 3.31. Scorzonera sublanata Lipsch.

Samples of *S. sublanata* were collected in Turkey and aerial parts and roots were separated [9,27]. The extraction was carried out using *n*-hexane [27] and 20% aqueous methanol [9].

Aerial part aqueous methanol extract contains chlorogenic acid and hyperoside [9]. In the *n*-hexane extract, the presence of lupeol, lupeol acetate, and taraxasteryl acetate was reported [27].

Root aqueous methanol extract was reported to contain chlorogenic acid [9]. The *n*-hexane extract is reported to contain taraxasteryl acetate, 3β-hydroxy-fern-7-en-6-one-acetate, lupeol, and lupeol acetate [27].

### 3.32. Scorzonera tomentosa L.

Samples of *S. tomentosa* were collected in Turkey [5,27,36]. Aerial parts and roots were separated and extracted using *n*-hexane [27], and 20% aqueous methanol [5]. Subaerial parts were extracted with methanol at room temperature [36].

Aerial part *n*-hexane extract is reported to contain lupeol, lupeol acetate, and taraxasteryl acetate [27]. In the aqueous ethanol extract, chlorogenic acid, hyperoside, and cyranoside were detected [5].

In the roots, the presence of taraxasteryl acetate, 3β-hydroxy-fern-7-en-6-one-acetate, olean-12-en-11-one-3-acetyl, lupeol, lupeol acetate, and α-amyrin was detected in the n-hexane extract [27]. The aqueous methanol extract was also reported to contain chlorogenic acid [5], (±)-scorzotomentosin, (‒)-,scorzotomentosin, (‒)-scorzotomentosin 4′-O-β-glucoside, (±)-scorzophtalide, scorzoerzincanin, (±)-hydrangenol, (‒)-hydrangenol 4′-O-glucoside, (±)-hydramacrophyllol A, and (±)-hydramacrophyllol B [36].

### 3.33. Scorzonera trachysperma Guss.

Aerial parts of *S. trachysperma* samples, collected in Italy, were extracted with 50% aqueous methanol and subjected to HPLC.

The analysis revealed that the aerial part methanol:water extract contains chlorogenic acid, *cis*-chlorogenic acid, cryptochlorogenic acid, isochlorogenic acid A, isochlorogenic acid C, 3,5-dicaffeoylquinic acid, isoorientin, luteolin, and apigenin diglycosides, and luteolin [26].

### 3.34. Scorzonera undulata ssp. alexandrina Boiss.

Samples of the plant were collected in Algeria and the whole plant was macerated with petroleum ether.

Lupeol, 24-methylenecycloartanol, 3-*O*-(6-*O*-acetyl-β-D-glucopyranosyl)-β-sitosterol, daucosterol, and apigenin were isolated from *S. undulata* ssp. *alexandrina* whole plant petroleum ether extract [23].

### 3.35. Scorzonera undulata ssp. deliciosa (Guss.) Marie

Plant samples were collected in Algeria and subaerial parts were macerated in dichloromethane, then the isolation of compounds was performed.

From the roots of the plant, the following compounds were isolated: verbascoside, galangustin [49], cichoriin, β-amyrin acetate, β-sitosterol, stigmasterol methyl oleanate, ethyl ursolate [63].

### 3.36. Scorzonera veratrifolia Fenzl.

The samples used for the studies on *S. veratrifolia* were collected in eastern Turkey. The subaerial parts were separated from the aerial parts and extracted with methanol at room temperature [30,64].

Subaerial parts of *S. veratrifolia* contain the following triterpenes: α-amyrin acetate, α-amyrinone, β-amyrin acetate, β-amyrinone, β-amyrin, ψ-taraxasterol, ψ-taraxasterol acetate, fern-7-en-3-one, germanicol, germanicol acetate, germanicone, lupenone, lupeol, lupeol acetate, taraxasterol, and taraxasterol acetate, as well as β-sitosterol [30]. The presence of chlorogenic acid, chlorogenic acid methyl ester, isochlorogenic acid A, cryptochlorogenic acid, 4,5-dicaffeoylquinic acid, together with scorzoveratrin and scorzoveratrozit has also been reported [64].

### 3.37. Scorzonera villosa Scop. ssp. villosa

Samples of *S. villosa* were collected in Slovenia. Aerial parts were extracted with 50% aqueous methanol at room temperature and HPLC analysis was performed.

The extract of aerial parts was reported to contain 1,5-*O*-dicaffeoylquinic acid, 3,5-*O*-dicafeoylquinic acid, 5-*O*-caffeoylquinic acid, chlorogenic acid, cryptochlorogenic acid, apigenin 7-*O*-glucuronide, apigenin di-*C*-glycoside, apigetrin, cyranoside, hyperoside, isochlorogenic acid C, isoorientin, isoquercitrin, luteolin, luteolin 7-*O*-glucuronide, and vitexin [26].

Compounds present in aerial parts of species within the genus *Scorzonera*, together with the concentration in the dry plant matter and the solvent used in the process of extraction (if available in the literature) are listed in Table 1. The phytochemical composition of subaerial parts of species belonging to the genus, with concentrations and solvents used, is presented in Table 2. Compounds isolated from the whole plants are presented in Table 3. As was presented in Table 1 and Table 2, subaerial parts of the species within the genus *Scorzonera* are reported to contain a greater diversity of triterpenoid ad phenolic acid derivatives. This could be explained by the fact that a larger range of solvents was used for the extraction of phytochemicals from subaerial parts. The aerial parts; however, are reported to contain notably more different flavonoids. This is an anticipated outcome because, as flavonoid compounds are involved in biochemical processes within the whole plant, they are significant for the activities related to exposure to external factors (e.g., UV radiation or attracting pollinators) [65,66]. Another reason is the fact that only aerial parts of *Scorzonera* species were thoroughly assessed for flavonoid content in a study by Granica et al. [26]. Based on the research included in this review, the steroid, coumarin, and dihydroisocoumarin content seems similar in both aerial and subaerial parts. The species that are most investigated in the greatest number of papers are *S. divaricata* [14,34,57,58,59], *S. hispanica* [20,26,32,45,46,60], *S. latifolia* [3,4,5,6,27,47,67,68,69,70,71], and *S. radiata* [12,13,15].

## 4. Biological Activity

The biological activity of species within the *Scorzonera* genus is the subject of research due to their presence in folk medicine in Eurasia and northern Africa. In Mongolia, *S. divaricata* and *S. pseudodivaricata* play a significant role in herbal therapy. *S. divaricata* is used to treat fever and poisonous ulcers or even malignant stomach neoplasia. *S. pseudodivaricata* is a folk remedy for digestive problems, parasites, or lung edema [14]. 

**Figure 1:**
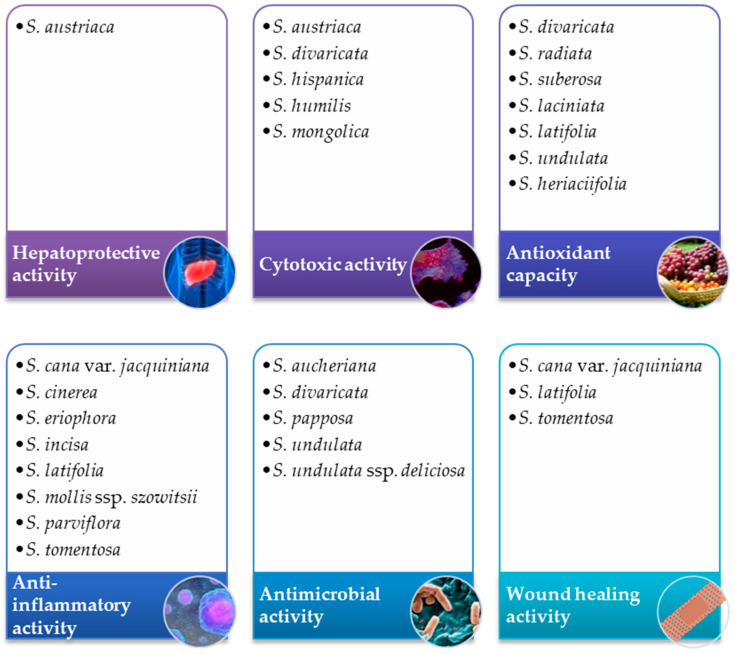
Biological activity of species within the genus *Scorzonera* evaluated in vitro. Species were assigned to sections according to their activity investigated in in vitro tests, described in Section 4.1, Section 4.2, Section 4.3, Section 4.4, Section 4.5, Section 4.6 and Section 4.7.

Species that belong to the *Scorzonera* genus are reported to be the source of numerous bioactive compounds. Researchers evaluate their potential as antioxidant [58,59,62,74], anti-inflammatory [27,68,75], and pain-relieving agents [6,70], as well as their cytotoxicity against cancer cell lines [20,28,59] and wound healing properties [4,5]. Biological activities of *Scorzonera* species in vitro are summarized in Figure 1.

### 4.1. Cytotoxic Activity

Cytotoxicity is the primary characteristic of compounds and substances in terms of their qualification as therapeutic agents and is the toxicity that a certain factor causes in live cells [76]. High cytotoxicity against rapidly dividing cancer cells in vitro is the basis for further research on their bioactivity (e.g., necrosis, autophagy, or apoptosis induction), low cytotoxicity on the other hand is desired in the development of drugs that are not intended to induce death in cells. In this review, the cytotoxicity of extracts and compounds obtained and isolated from *Scorzonera* species against cancer cell lines was presented.

The first reported attempt to evaluate the antineoplastic activity of *Scorzonera* species in vitro was made in 2000 by Zidorn and colleagues [54]. The biological activity of compounds isolated from a methanol extract from *S. humilis* subaerial parts (together with newly isolated tyrolobibenzyls) was assessed and none influenced the DNA biosynthesis in the GTB and HL60 human leukemia cell lines at the concentration range of 0.25–4.00 μM. In their further research, tyrolobibenzyls D was isolated from the extract. It was assayed for cytotoxicity against the P388 (mouse leukemia) cell line along with previously obtained tyrolobibenzyls A-C and their peracetyl derivatives. In the assay, only Tylorobibenzyl D exhibited low cytotoxic activity with IC_50_ (half-maximal inhibitory concentration) of 25 μg/mL. The cytotoxicity of crude extracts was assayed as well and no activity was observed up to the point where the concentration reached 0.5 mg/mL. The EtOAc fraction of the crude extract exhibited cytotoxic properties with IC_50_ value at the concentration of 95 μg/mL. In the research, the antimicrobial activity of tyrolobibenzyls and their derivatives was evaluated but none was active against neither bacteria nor fungi. The DPPH assay did not reveal any significant radical scavenging properties of tyrolobibenzyls and the compounds were able to inhibit the activity of COX-1 (cyclooxygenase 1) to an insignificant degree [53].

In the study from 2009, Wang and colleagues [31] obtained two triterpene fatty esters: erythrodiol and 3β-tetradecanoyl erythrodiol from a methanol extract of *Scorzonera mongolica* whole plants. The isolated compounds were then assayed for their cytotoxicity towards 3 cancer cell lines (P388 mouse leukemia cell line, A549 human lung cancer cell line, and Bel-7402 human hepatocellular carcinoma cell line) and both esters exhibited cytotoxic activity against A549 lung cancer cells (in the concentration of 50 μg/mL, the compounds induced the cell growth inhibition by 66.8% and 69.8%).

Two out of five congeners (Scorzodihydrostilbenes A and B) isolated from the methanol extract from the aerial parts of *Scorzonera radiata* Fisch. were tested in the MTT cytotoxicity assay but at a concentration of 10 µg/mL, neither displayed cytotoxic activity toward mouse lymphoma cell line (L5175Y) [15]. The concentration (10 μg/mL) converted to μM is 21.53 μM for Scorzodihydrostilbene A and 20.90 μM for Scorzodihydrostilbene B. When compared to a study from 2007, dihydrostilbenes isolated from a *Bulbophyllum odoratissimum* Lindl., low toxicity of *Scorzonera radiata* Fisch. is even more notable, compounds in the mentioned study were toxic towards SGC-7901 (human gastric cancer), KB (nasopharyngeal carcinoma), and HT-1080 (fibrosarcoma) cell lines with IC_50_ values of 5.50–9.20 μM for SGC-7901 and KB lines and 25.50–40 μM for HT-1080 line [77].

A screening study on cytotoxic activity of several species of *Asteraceae* genus present in Hungary, including *Scorzonera austriaca* Willd, was carried out in 2009. In a cytotoxicity assay, the most active against human cell lines: A431 (skin epidermoid carcinoma), HeLa (cervix adenocarcinoma), and MCF-7 (breast adenocarcinoma) was a chloroform root extract of *S. austriaca* IC_50_ values of the extract were: 4.71 μg/mL for A431 line, 6.42 μg/mL for HeLa line and 5.52 μg/mL for MCF-7 line. It was more active than the extracts obtained using other solvents, as well as leaf extracts from the same plant—at a concentration of 10 μg/mL the antiproliferative activity of *S. austriaca* root chloroform was at 86.32% for A431 cell line, 77.27% for HeLa cell line, and 83.79% for MCF-7 cell line. Other *S. austriaca* extracts obtained in the study did not influence the proliferation of those cell lines by more than 48.11% [78]. Those results can be compared to a 2018 research, in which leaf chloroform extract from another species within the *Asteraceae* family, *Anvillea garcinii* (Burm.f.) DC., exhibited antiproliferative properties against MCF-7 and HeLa cell lines with IC_50_ of 24.50 μg/mL for MCF-7 and 12 μg/mL for HeLa [79]. Another *Asteraceae* family member, *Pulicaria undulata* (Forssk.) Oliver., was evaluated as a potential source of cytotoxic agents. Whole plant chloroform extract turned out to have cytotoxic properties with the IC_50_ value of 16.4 μg/mL for MCF-7 cell line, 3.01 μg/mL for HepG2, and 7.4 μg/mL for HCT-116 cell lines. Those values were compared to cisplatin used as a positive control in the study (IC_50_ of cisplatin was 3.68–4.51 μg/mL) [80]. However, in the study from 2011, Bader et al. isolated nine new phenolic compounds and nine known phenolic derivatives from *Scorzonera judaica* root extracts. The newly obtained compounds were assayed for their cytotoxic activity toward human lymphocyte T cells, as well as MCF-7 and HeLa cell lines. Compounds did not exhibit cytotoxic activity in concentrations below 100 µM [25].

Zhu and colleagues [17] discovered that two new dimeric guaianolides (biguaiascorzolides A and B) are present in an acetone extract from *Scorzonera austriaca* roots. In the study, biguaianoscorzolide A was acetylated and the derivative’s cytotoxicity against adriamycin-resistant myelogenous leukemia (K562/ADM) cell line and gastric carcinoma (BGC-823) cell line was measured. The compound’s activity towards K562/ADM cells (IC_50_ = 39.8 μM) was more significant than towards the MGC-803 cell line (IC_50_ > 100 μM), which suggests that the compound’s cytotoxic activity might depend on the type of tumor cells. In a continuation of the study, Zhu and colleagues [33] elucidated the presence of six sesquiterpene lactones in acetone and ethanol *S. austriaca* root extracts (scorzoaustriacoside, scorzoaustriacin, scorzoaustriacin 3-*O*-β-D-glucoside, 4-*epi*-dihydroestafiatol, 14-isovaleroxyscorzoaustricin, and 14-isovaleroxyscorzoaustricin sulfate). The cytotoxic activity against four cancer cell lines (K562, K562/ADM, BGC-823, and Hep-G2) of scorzoaustriacin, scorzoaustriacin 3-*O*-β-D-glucoside, 14-isovaleroxyscorzoaustricin, and 14-isovaleroxyscorzoaustricin sulfate was assayed and only scorzoaustriacin was reported cytotoxic towards K562 (human myelogenous leukemia) cell line (IC_50_ = 11.3 μM).

In the study by Granica et al. [20], (–)-syringaresinol was isolated from the ethyl acetate extract of *Scorzonera hispanica* subaerial parts. It was then reported that (–)-syringaresinol was cytotoxic towards NCI and MMS-1 myeloma cell lines and exhibited moderate activity against SW-480 colon cancer cells. Moreover, the compound’s cytotoxicity was reported in peripheral blood mononuclear cells. In the previous research on (–)-syringaresinol, it was reported to exhibit the ability to induce apoptosis and arrest the G_1_ phase in the HL-60 human leukemia cell line [81]. Jeong et al. [82] found out that (–)-syringaresinol inhibits P-glycoprotein in MCF-7/ADR human breast cancer cell line and enhances the cytotoxic activity of daunomycin.

In a phytochemical study on *Scorzonera divaricata* root ethanol extract, one of the isolated compounds, a tirucallane terpene ((3*S*,5*R*,10*R*,13*S*,14*S*,17*R*,20*S*,24*R*)-3-acetoxy-24-hydroxyl-tirucalla-7,25-dienem, named scorzodivaricin B), exhibited a cytotoxic activity towards HeLa, HepG2, HL60 and SMMC-7721 (human cervical cancer, human liver cancer human leukemia cancer and hepatocellular carcinoma, accordingly) cell lines with IC_50_ values between 24.4 ± 3.6 μM and 66.7 ± 5.2 μM. Cisplatin used as a positive control in the study exhibited higher activity with IC_50_ within the range of 7.45 ± 0.9–12.8 ± 2.4 μM [59].

Wu and colleagues [34] evaluated five compounds isolated from *Scorzonera divaricata* aerial parts for their potential activity against cancer cell lines (HepG2, HeLa, and K562). Sulfoscorzonin E exhibited cytotoxicity similar to 5-FU. IC_50_ values of sulfoscorzonin E were 4.21 μg/mL (10.59 μM) for HepG2, 8.15 μg/mL (20.5 μM) for HeLa, and 6.53 μg/mL (16.43 μM) for K562 cell line. Sacrolide A was active against HeLa and HepG2 cells (IC_50_ = 6.28 μg/mL = 20.16 μM for HeLa and 3.56 μg/mL = 11.43 μM for HepG2 lines). In the study, sulfoscorzonin D, glucozaluzanin C, and 1β,4α-dihydroxy-5α,6β,7α,11β*H*-eudermn-12,6-olide did not exhibit any significant cytotoxic activity at the concentration below 80 μg/mL (141.92–298.32 μM).

### 4.2. Anti-Inflammatory Activity

For centuries, suppression of inflammatory response has been an observed effect of various medicinal plants. Ethnopharmacological reports give examples of plants extracts able to combat the process of inflammation in human bodies and; therefore, novel plant-derived products are investigated for their anti-inflammatory activity [83].

A study carried out by Bahadır-Acıkara et al. in 2018 [27] revealed that *n*-hexane extracts from roots and aerial parts of eleven *Scorzonera* species (*S. acuminata*, *S. cinerea*, *S. eriophora*, *S. incisa*, *S. latifolia*, *S. mirabilis*, *S. mollis* ssp. *szowitsii, S. parviflora*, *S. suberosa* ssp. *suberosa* and *S. tomentosa*) contained significant amounts of triterpenes, including taraxasteryl acetate, lupeol, and lupeol acetate. In general, root extracts were notably richer in analyzed triterpenes, except for lupeol, whose concentration in aerial parts of *S. incisa*, *S. latifolia*, *S. mirabilis, S. parviflora,* and *S. suberosa* was approximately three to seven times higher than in root extracts (approximately 0.9–1.5 mg/g)^.^ The results from the study correlate with previously observed anti-inflammatory and pain-relieving properties of lupeol [84,85].

The evaluation of the anti-inflammatory properties of *Scorzonera pygmaea* subaerial parts was conducted in 2018 by measuring COX (cyclooxygenase) inhibition. The inhibitory activity of ethanol extract and its fractions against COX-1 and COX-2 (cyclooxygenase 2) was low [35]. That is on the contrary to the study of Bahadır Acıkara et al. from 2015 [10], where it has been observed that extracts from other *Scorzonera* species (*S. cana* var. *jacquiniana, S. cinerea, S. eriophora, S. incisa, S. latifolia, S. mollis* ssp. *szowitsii, S. parviflora,* and *S. tomentosa)* have an inhibitory effect on pro-inflammatory cytokines (TNF-α (tumor necrosis factor α) and IL-1β (interleukin 1β)) production and NF-κB (nuclear factor kappa B) nuclear translocation in macrophages. However, it might suggest that the anti-inflammatory activity of *S. pygmaea* could be evaluated by the measurement of the inhibitory activity against pro-inflammatory cytokines, as they induce COX production [35].

### 4.3. Analgesic Activity

Pain is an experience known to nearly every animal. There are cases where pain requires medical intervention. Although pain may indicate injury of organs, nervous system-derived pain should be relieved beforehand to prevent the deterioration of the quality of the patient’s life. Morphine, a well-known analgesic, was isolated from opium 200 years ago [86]. Nowadays other natural products are assayed for their pain-relieving activity.

*Scorzonera latifolia* is a plant endemic to Turkey, whose roots are used as a pain-reducing and anthelmintic agent in Turkish folk medicine [4]. An in vivo study on the properties of a methanol extract from *S. latifolia* roots showed that *n*-hexane, chloroform, ethyl acetate, *n*-butanol, and water fractions indeed exhibit analgesic activity on mice in the dose of 50 mg/kg in the tail-flick test. Taraxasteryl mirystate and taraxasteryl acetate present in one of the extracts were active in the dose of 10 mg/kg in both writhing test and flick-tail test. The general antinociceptive properties of *S. latifolia* are reported to be significant. Such outcome of the study can be a result of the synergy of the extract’s components [6]. The study was extended to four *Scorzonera* species in 2012. It was then reported that *S. tomentosa, S. latifolia,* and *S. mollis* ssp. *szowitsii* all possess analgesic properties in the writhing test and tail-flick test (the dose was 100 mg/kg) [70]. 

### 4.4. Hepatoprotective Activity

The liver plays a significant role in the metabolism and detoxication of the human body. Because of its importance, liver diseases are one of the greatest threats to people’s lives. Herbal medicine has used plants as preventive agents for hepatic problems for ages [87]. Based on that knowledge, in vitro and in vivo investigations are conducted to assess the hepatoprotective potential of plants, including the ones with the *Scorzonera* genus.

#### 4.4.1. In Vitro Assays

A study from 2016 investigated the hepatoprotective properties of *Scorzonera austriaca*. The plant is used in folk medicine to treat hepatitis B in China. Xie et al. [24] isolated flavonoid glycosides and their derivatives from *Scorzonera austriaca* herb ethanolic extract. Having measured the concentration of ALT (alanine aminotransferase) in CCl_4_-treated rat hepatocytes, it was reported that two flavonoid glycoside derivatives, 5,7,41-trihydroxyflavone 8-*C*-(6″-*O*-*trans*-caffeoyl β-D-glucopyranoside) and 5,7,31,41-tetrahydroxyflavone 8-*C*-(6″-*O*-*trans*-caffeoyl β-D-glucopyranoside), present in herbs of *S. austriaca* have hepatoprotective properties. That conclusion confirmed the validity of the use of the plant in the treatment of hepatitis B in the traditional medicine of China.

#### 4.4.2. In Vivo Assays

The in vivo assays of a *Scorzonera alexandrina* hydroethanolic extract from aerial and subaerial parts revealed that the extract caused a reduction in glucose concentration in rat’s blood, as well as the ALT, and total protein levels in doses of 200 and 400 mg/kg. The extract also exhibited hepatoprotective, and anti-ulcerogenic effects in rats [75].

Hepatoprotective activities of the roots of several *Scorzonera* species (*S. cana* var. *jacquiniana, S. latifolia*, *S. mollis* ssp. *szowitsii*, *S. parviflora, S. tomentosa*), together with compounds isolated from the *S. latifolia* root extract (chlorogenic acid, hydrangenol-8-O-β-glucoside, and scorzotomentosin-4′-O-β-glucoside) were evaluated in a preclinical in vivo study from 2017. The tests were aiming to elucidate the extract’s influence on counteracting CCl_4_-induced liver damage in rats. Although the influence of the extract and compounds on the ALT and AST (aspartate transaminase) levels was insignificant, the histological condition of animal livers was notably better in most samples (except for hydrangenol-8-*O*-β-glucoside and scorzotomentosin-4′-*O*-β-glucoside—treated groups). What is interesting in terms of future clinical research is the fact that chlorogenic acid was the most active compound in the treatment of acute carbon tetrachloride-induced liver toxicity [73].

### 4.5. Antimicrobial Activity

Folk medicine has been treating microbial infections for centuries. Along with the decrease in bacteria’s susceptibility to antibiotics, the need for novel antimicrobial drugs is increasing. Plants have been a source of folk medications in the treatment of infectious diseases before the concept of infectious agents emerged [88]. The activity against microbes could also be used to substitute synthetic additives in food to prevent foodborne diseases induced by pathogenic bacteria [89] Several species within the genus *Scorzonera* have been investigated as a source of products with antimicrobial potential.

Volatile oil distilled from aerial parts of *Scorzonera undulata* ssp. *deliciosa* was assessed as an antibacterial agent against Gram-positive (*Staphylococcus aureus*, *Staphylococcus epidermidis,* and *Micrococcus luteus*) and Gram-negative (*Salmonella typhimurium*, *Escherichia coli,* and *Pseudomonas aeruginosa*) bacteria strains and it was reported more active towards Gram-positive strains with MIC (minimal inhibitory concentration) values of 0.5 mg/mL for *S*. *aureus* and *M*. *luteus* and 0.8 mg/mL for *S. epidermidis*, *S. typhimurium,* and *E. coli*. MBC (minimal bactericidal concentration) was not determined for any Gram-negative strain. The authors suggest that greater activity against Gram-positive bacteria strains could be caused by easier penetration through the lipophilic cell membranes by hydrophobic ingredients of the oil. Unfortunately, no reference compound was assessed together with the oil, thus it is difficult to compare those results with any known antibacterial substances [90]. 

Antibacterial properties of *S. undulata* were assayed in 2010 by Abdelkader and colleagues [91]. The study showed that ethyl acetate fraction of the aerial part methanol extract exhibited antibacterial properties against *P*. *aeruginosa*, *S*. *aureus*, *E*. *faecalis*, *C*. *freundei,* and *P*. *mirabilis* with MIC exceeding 1 mg/mL. The petroleum ether fraction; however, was active against *P*. *aeruginosa*, *S*. *aureus,* and *C*. *freundei.* Fractions obtained from the roots had a narrower spectrum of activity, but the petroleum ether fraction exhibited a stronger antimicrobial potential against *S. aureus* with a MIC of 500 μg/mL.

Bactericidal properties of compounds isolated from a *Scorzonera divaricata* aerial parts petroleum ether/diethyl ether/methanol extract were evaluated in a study by Wu and colleagues in 2018 [34]. Sulfoscorzonin D, a new, rare pyrrolidine salt alkaloid obtained in the study, exhibited more potent activity against *Clostridium perfingens* than ampicillin, its activity was similar to the activity of erythromycin and streptomycin. Sacrolide A was more effective against Newman WT than streptomycin (12.5 to 25 μg/mL) and similarly effective to levofloxacin (12.5 μg/mL). Other compounds which were reported to possess antibacterial properties (*B. megaterum*, *C. perfingens*, Newman WT, and *E. coli*) were oleanolic acid, lup-20(29)-ene3β,28-diol, (22E)-5α,8α-epidioxyergosta-6,22-dien-3β-ol, ergosta-3β,5α,6β-trialcohol, and diosmetin (MIC values were 12.5–100 μg/mL).

A comprehensive study on endemic to Lebanon species *Scorzonera mackmeliana* in terms of the plant’s antibacterial and antibiofilm properties was carried out by Sweidan and colleagues in 2020 [92]. The authors conducted a phytoanalysis of the constituents of water and ethanol extracts of the whole plant as well as its particular parts (flowers, stems, leaves, roots) and determined their activity against Gram-positive (*Staphylococcus epidermidis*, *Staphylococcus aureus*, *Enterococcus faecalis*) and Gram-negative (*Escherichia coli*, *Pseudomonas aeruginosa*) bacterial strains. The highest activities in inhibition of the bacteria strains were observed in two water extracts: the ones obtained from the stems and the whole plant. For the stem water extract, the inhibitory effect was observed in four out of five tested strains, the lowest MIC values were reported in *P. aeruginosa* (48.98 mg/mL), *S. aureus* (48.98 mg/mL), and *S. epidermidis* (48.98 mg/mL). For the water extract from the whole plant, it was proven active against *S. epidermidis*, *P. aeruginosa,* and *E. coli* in the concentration of 122.25 mg/mL for each strain. MBC was determined for only two extracts and those were root ethanol extract (for *S. epidermidis* MBC = 284.35 mg/mL) and water flower extract (for *P. aeruginosa*, MBC = 160.85 mg/mL). Those values; however, are notably high, compared to the literature data available for other plant water and ethanol extracts. MIC and MBC values for *Cinnamomum impressicostatum* stem bark extract against MRSA strain were 19.53 and 39.06 μg/mL, respectively. For *Cinnamomum porrectum* stem bark extract, it was 2.5 (MIC) and 5 mg/mL (MBC) [93]. In the *Asteraceae* family, water extracts from *Sonchus erzincanicus* aerial parts exhibit antibacterial properties against *Staphylococcus aureus*, *Escherichia coli*, and *Proteus mirabilis* with the MIC value of 1.25 mg/mL [94]. In the mentioned study on *S*. *mackmeliana;* however, an interesting pattern of the activity against biofilm formation was observed—with the concentration decrease, greater destruction of the biofilm occurred. It was also observed that flower and stem water extracts and ethanol leaf, flower, and whole plant extracts exhibited the most potent activity in eradicating bacterial biofilm, with MBEC (minimal biofilm eradication concentration) values of 0.1–2.2 mg/mL, causing 84–98% biofilm eradication. For water extracts, the presence of coumarin was suspected to be the active factor and in ethanol extracts, terpenoids were major constituents and; therefore, they are thought to be responsible for the antibiofilm effect [92].

The antibacterial and antifungal activity of the aerial parts and root extracts from *S. papposa* was investigated in a recent study by Mohammed and colleagues [95]. The extracts were toxic for bacteria at the concentrations of 50–800 µg/mL and their antifungal effectiveness was observed at 50–100 µg/mL. The reference compounds (ampicillin, amikacin, ciprofloxacin, fluconazole, and amphotericin B) on the other hand were active at notably lower concentrations (1.56–3.12 μg/mL). In general, samples collected in Turkey were slightly more effective against both bacteria and fungi (50 μg/mL against *Pseudomonas aeruginosa*), although all tested extracts can potentially be used as mild nature-derived antimicrobial agents.

In a study on antimicrobial activities of compounds isolated from aerial parts of *Scorzonera aucheriana* it was observed that scorzoaucherioside II, iso-scorzopygmaecoside, and 3,4-dihydroxyphenyl caffeate possess strong anti-tuberculosis activity with MIC of 21.2 μg/mL, 25.6 μg/mL, and 145 μg/mL, respectively. 3,4-Dihydroxyphenyl caffeate and scorzoaucherioside I were reported active against Gram-negative bacteria strain *Pseudomonas aeruginosa* (MIC = 290 and 377.5 μg/mL, respectively). *Enterococcus faecalis*, a Gram-positive strain, was reported sensitive to scorzopygmaecoside and scorzocreticoside II, where MIC values were 135 and 200 μg/mL, respectively [7].

### 4.6. Wound Healing Activity

Wounds defined as a disruption of tissue do not pose a threat unless the blood loss is significant. They can; however, be a gateway for pathogenic infections which is much more dangerous for the patient. The acceleration of wound recovery includes infection prevention and the promotion of the natural healing process. Medicinal plants are suspected to possess both those qualities and are considered potentially effective in the therapy of wound healing [96].

#### 4.6.1. In Vitro Assays

A study carried out by Küpeli Akkol and colleagues in 2019 [4] reports that ethyl acetate and chloroform fractions of a methanol extract from aerial parts of *S. latifolia* have wound healing properties in vitro, which are a result of the inhibitory effect on collagenase and elastase enzymes activity. None of the fractions influenced the activity of hyaluronidase to any considerable degree.

In a study on the extract from *S. cana* var. *jacquiniana* aerial parts (stems, leaves, and flowers), their activity against matrix metalloproteinases (collagenase, hyaluronidase, and elastase) was evaluated. A methanol extract was partitioned into chloroform, petroleum ether, ethyl acetate, and water fraction. From the ethyl acetate fraction, eleven compounds were isolated (3,5-dicaffeoylquinic acid methyl ester, 4-hydroxy-benzoic acid 4-(6-*O*-α-rhamnopyranosyl-β-glucopyranosyl) benzyl ester, 6′-*O*-caffeoylarbutin, apigenin 7-*O*-β-glucoside, apigenin 7-*O-*β-rutinoside, arbutin, cichoriin, isoorientin, luteolin 7-O-β-glucoside, orientin, protocatechuic acid, and vitexin) and all obtained samples were tested for their enzyme-inhibitory properties. In the hyaluronidase inhibition assay, the extracts, fractions, and isolated compounds exhibited only a mild inhibitory effect at the concentration of 100 μg/mL (not exceeding 30% for luteolin 7-*O-*β-glucoside), compared to the positive control—Tannic acid—Which inhibited the hyaluronidase activity by approximately 75%. The methanol extract was observed to possess a potent inhibitory effect on both elastase and collagenase activity (51.7% inhibition for elastase and 35.7% for collagenase, in the concentration of 100 μg/mL). Moreover, several compounds isolated from the extract were significantly active against collagenase (apigenin 7-O-β-glucoside, apigenin 7-*O-*β-rutinoside, and isoorientin) and elastase (apigenin 7-*O-*β-glucoside, luteolin 7-*O-*β-glucoside, and apigenin 7-*O-*β-rutinoside) as well. In light of those results, the authors suggested that flavonoids present in methanol extract were the main wound-healing agents and that the synergy between components of the extract contributes to the inhibitory effect [72].

#### 4.6.2. In Vivo Assays

In 2011, a study by Küpeli Akkol et al. [5] on the promotion of the process of wound healing in mice took place. Researchers obtained hydroethanolic extracts from the aerial parts and roots of several *Scorzonera* species (*S. cinerea*, *S. latifolia*, *S. incisa*, *S. mobilis*, *S*. *mollis* ssp. *szowitsii*, *S. tomentosa*). Most promising results in the assays carried out with wound models were observed with ointments made with the extracts obtained from *S. latifolia*, *S. mollis* ssp. *Szowitsii,* and *S. tomentosa* aerial parts. Those three extracts were the most active in the hydroxyproline level enhancement as wells as in terms of skin remodeling. Moreover, the *S. latifolia* aerial part extracts were reported to have anti-inflammatory properties in vivo, with an inhibitory value of 23.5% at the dose of 100 mg/kg.

A study from 2012, carried out on mice, reports wound healing properties of several *Scorzonera* species. Aqueous methanolic extracts from the aerial parts of *S. cana var. jacquiniana*, *S. eriophora,* and *S. acuminata* caused the contraction of wound area by up to 46.27% on day 12 in the circular excision wound model. Ointments containing extracts of *S. cana* (C.A. Mey.) Hoffm. var. *jacquiniana* (W. Koch) Chamb. and *S. eriophora* DC. aerial parts, when applied topically on the linear incision wound models, caused an increase in the activity of anti-hyaluronidase and significant enhancement of hydroxyproline level in the regenerated tissue [9].

**Figure 2:**
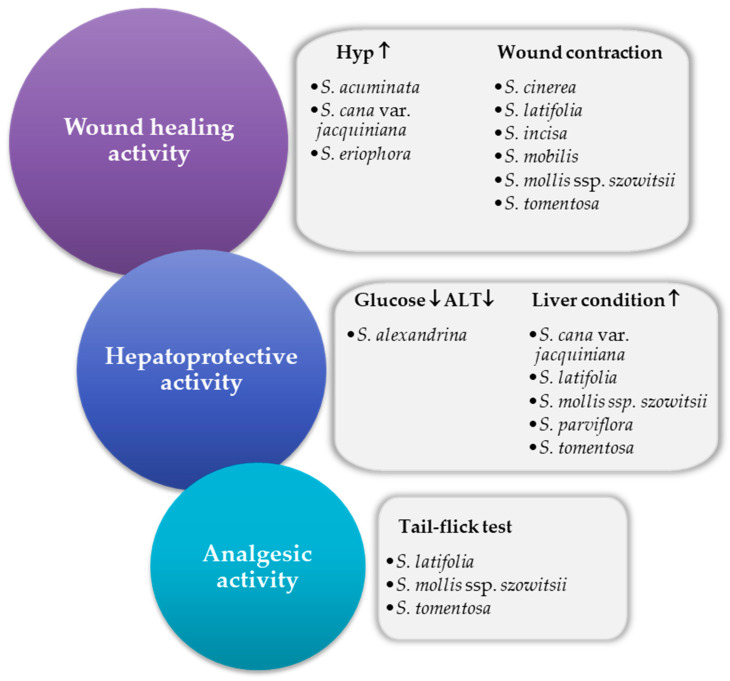
Biological activity of species within the genus *Scorzonera* evaluated in vivo. Hyp↑: Increase in the hydroxyproline level; Glucose↓: Decrease in the glucose level; ALT↓: Decrease in the alanine transaminase level; Liver condition↑: Improvement in the liver condition.

Figure 2 summarizes literature data regarding the biological activity of *Scorzonera* species in vivo.

### 4.7. Antioxidant Capacity

Exposure to reactive species in the environment may have a negative impact on humans and animals. The balance between oxidants and antioxidants is becoming more difficult to maintain [97].; therefore, the need for antioxidant agents is growing and, because of the potentially harmful effect of synthetic antioxidants, the attention seems to be currently directed towards naturally occurring antioxidants found in plants [98]. The products from species belonging to the genus *Scorzonera* have been assayed as antioxidant agents as well.

In the DPPH radical scavenging assay compounds isolated from the ethyl acetate fraction of the methanol extract from *Scorzonera divaricata* and *Scorzonera pseudodivaricata* aerial parts (ferulopodospermic acid A and B) did exhibit a strong antioxidant activity, more potent than chlorogenic acid used as a reference compound in the study. The IC_50_ values ferulopodospermic acid A and B were 36.36 and 34.24 μmol/mL, respectively, compared to the IC_50_ of chlorogenic acid, which was 67.92 μmol/mL [14]. The study was continued with five compounds obtained from a *Scorzonera radiata* aerial part MeOH extract were tested for their radical-scavenging activity in the DPPH assay. Scorzodihydrostilbenes A and E exhibited a higher activity level than resveratrol, well-known for its antioxidant activity, used as a reference in the study. The IC_50_ values were 105.51 μM for scorzodihydrostilbene A, 102.60 μM for scorzodihydrostilbene B, and 149.52 μM for resveratrol [15]. Although the difference in the scale of IC_50_ values might seem interesting, as IC_50_ in *S*. *divaricata* and *S*. *pseudodivaricata* was given in μmol/mL, whereas for *S*. *radiata* it was presented in μM (μmol/L), more informative is how those results correspond to reference compounds used in both studies.

In the assessment of antioxidant activity, acteoside isolated from a methanolic extract from *Scorzonera undulata* ssp. *deliciosa* roots was reported to possess similar antiradical power to Trolox used as a standard in the DPPH test (IC_50_ values were 0.16 ± 0.02 mg/mg DPPH for acteoside and 0.2 ± 0.01 mg/mg DPPH for Trolox). The Trolox Equivalent Antioxidant Capacity (TEAC) value of acteoside in the DPPH assay was 1.25. In the CUPRAC (cupric reducing antioxidant capacity) assay acteoside was slightly less active than the reference compound, rutin (TEAC = 3.16 for rutin compared to TEAC = 2.4 for acteoside) [49]. 

Nasseri et al. [52] evaluated the chemical composition and the radical scavenging activity of *Scorzonera paradoxa* root and leaf ethanol/water extracts. Leaf extracts turned out to be a more potent antioxidant with an IC_50_ value of 18.81 mg/mL, compared to the roots (IC_50_ = 88.9 mg/mL). This may be due to higher levels of phenolic compounds, flavonoids and tannins reported in the study. The authors also made an assessment of the fatty acids composition of the plant samples and based on the data obtained in the study of chemical composition and antioxidant properties, it was suggested that *S. paradoxa* might be successful as an antidiabetic agent. Those results and IC_50_ values correspond with a study from 2013, when Erden and colleagues [22] investigated the antioxidant properties of methanol extracts obtained from the leaves of three *Scorzonera* species (*S. suberosa*, *S. laciniata,* and *S. latifolia*). Those properties were examined in the DPPH assay and exhibited a concentration-dependent antioxidant activity with IC_50_ values of 29.36 mg/mL for *S. latifolia*, 42.33 mg/mL for *S*. *suberosa,* and 77.07 mg/mL for *S*. *laciniata*.

In 2013 Milella et al. [62] measured the antioxidant properties of pure compounds isolated from methanol extracts from aerial parts and roots of *Scorzonera papposa*. The authors assessed the antioxidant activity of the compounds obtained from *S. judaica* in Bader’s previous study from 2011 [25] as well. Four out of nine compounds isolated from *S. papposa* extracts were previously unknown. In the study, the antioxidant activity was measured in four different assays: the DPPH assay, the FRAP (ferric reducing antioxidant power) assay, the BCB (β-Carotene bleaching) assay, and the TPC (total phenolic content) assay. It has been observed that the antioxidant capacity of particular compounds depends on the method. The authors suggested that the antioxidant activity of the compounds found in the extract is a result of the synergistic effect of their combination. In the study, a new concept for presenting the antioxidant capacity of compounds—Relative Antioxidant Capacity Index (RACI)—Was applied [62]. Briefly, the parameter is used to integrate the data from several methods for the assessment of the antioxidant activity, where each method is assigned equal weight. RACI can take positive or negative values [99].

Yang and colleagues [59] assessed the antioxidant properties of several compounds isolated from an ethanol *Scorzonera divaricata* root extract. In the ABTS antioxidant capacity assay, two compounds obtained in the study—(*1R*,5*S*,6*S*,7*R*,8*S*)-8-sulfoxyguaia-4(15),10(14),11(13)-trine-6,12-olide (sulfoscorzonin A) and (1*R*,5*S*,6*S*,7*R*,8*S*,13*S*)-8-sulfoxy-13-L-prolineyl-guaia-4(15),10(14)-dien-6a,12-olide (sulfoscorzonin C)—Were reported to be moderately active in radical scavenging. The SC_50_ (half-maximal scavenging concentration) values were equal to 32.88 μM for sulfoscorzonin A and 24.86 μM for sulfoscorzonin C.

A recent study on *Scorzonera papposa* was a comparison of the antioxidant and antimicrobial activity of ethanol extracts from aerial parts and roots of *S. papposa* from Iraq and Turkey. It has been observed that samples from Iraq exhibited a higher level of TAS (Total Antioxidant Status) and a lower level of TOS (Total Oxidant Status) than samples collected in Turkey. Therefore, the OSI (Oxidative Stress Index—The TAS/TOS ratio) parameter in the samples from Iraq was lower than in the samples from Turkey. Compared to the reported TAS and TOS values of other plant species (i.e., *Calendula officinalis* L., *Rhus coriaria* L. var. *zebaria,* Shahbaz and *Mentha longifolia* L.), the extracts from the aerial parts of *S. papposa* obtained in the study exhibited a notable antioxidant activity [95].

## 5. Conclusions

Aerial and subaerial parts of species within the *Scorzonera* genus have been the subject of research regarding their phytochemical composition as well as their therapeutic potential. In many European and Asian cultures, *Scorzonera* species are commonly used in folk medicine,; therefore, modern phytoanalyses and biological studies have been carried out to verify the bioactive activities of the plants. Due to the presence of numerous bioactive compounds, including flavonoid aglycones and glycosides, triterpenoids, sesquiterpenoids, quinic acid, and caffeic acid derivatives, in the studied plant material, *Scorzonera* species are considered a potential source of antioxidant agents. Although the reported cytotoxicity of *Scorzonera* extract against cancer cell lines so far was insignificant, they exhibit other bioactive properties, potentially applicable not only in the therapy of pain, inflammation, and microbial infections, but also as an enhancement of the effectiveness of the wound healing process. It should be pointed out that a promising direction of further research on the genus *Scorzonera* is the investigation of their activity towards normal cell lines, especially skin cells, to assess their potential as wound-healing and skincare active agents.

## Figures and Tables

**Table 1:** Compounds present in aerial parts of *Scorzonera* species.

Compounds	*Scorzonera* Species	Concentration	Type of Solvent	References
**Benzoic acid derivatives**				
Methyl-3,4-dihydroxybenzoate	*S. divaricata*	0.6 μg/g	methanol	[34]
3-Hydroxybenzoic acid	*S. divaricata*	0.32 μg/g	methanol	[34]
**Coumarins and coumarin glycosides**				
Scopoletin	*S. divaricata*	3 μg/g	methanol	[34]
	*S. pseudodivaricata*	N/D	methanol	[14]
Hydrangenol 8-O-glucoside	*S. latifolia*	N/D	20% aqueous methanol	[10]
7-Hydroxycoumarin	*S. divaricata*	0.13 μg/g	methanol	[34]
**Dihydroisocoumarins and dihydroisocoumarins glycosides**				
(3S)-6-[O-β-d-glucopyranosyl-(6→1)-O-β-d-apiofuranosyl]-8-hydroxy-3-(4-methoxyphenyl)-3,4-dihydro-1*H*-isochromen-1-one (*iso*-scorzopygmaecoside)	*S. aucheriana*	19.208 μg/g	methanol	[7]
(3S)-6-{*O*-β-d-glucopyranosyl-[(4→2)-O-glyceryl)]-(6→1)-O-β-d-apiofuranosyl}-8-hydroxy-3-(4-methoxyphenyl)-3,4-dihydro-1*H*-isochromen-1-one (scorzoaucherioside I)	*S. aucheriana*	49.67 μg/g	methanol	[7]
(3S,3′ R)-8-{*O*-β-d-glucopyranosyl-[(4→2)-*O*-glyceryl)]-(6→1)-*O*-β-d-apiofuranosyl}-3-(4- methoxyphenyl)-6-{[3-(4-methoxyphenyl)-1-oxo-8-[*O*-β-d-glucopyranosyl-(6→1)-O-α-l-rhamnopyranosyl-(4→1)-O-β-d-glucopyran-osyl]-3,4-dihydro-1*H*-isochromen-6-yl]oxy}-3,4-dihydro-1*H*-isochromen-1-one (scorzoaucherioside II)	*S. aucheriana*	11.165 μg/g	methanol	[7]
Scorzopygmaecoside	*S. aucheriana*	9.724 μg/g	methanol	[7]
Scorzocreticoside II	*S. aucheriana*	5.642 μg/g	methanol	[7]
**Flavonoids**				
**Flavonoid aglycones**				
5,7-dihydroxy-6-methoxyflavone	*S. divaricata*	0.56 μg/g	methanol	[34]
5,7-dihydroxy-8-methoxyflavone	*S. divaricata*	2.01 μg/g	methanol	[34]
7,3′,4′-trihydroxyflavonol	*S. divaricata*	0.23 μg/g	methanol	[34]
Apigenin	*S. divaricata*	N/D	methanol	[44]
	*S. laciniata*	N/D	ethanol	[14]
Apigenin derivative	*S. austriaca*	N/D	methanol:water (1:1, *v*/*v*)	[26]
	*S. baetica*	N/D	methanol:water (1:1, *v*/*v*)	[26]
	*S. crispatula*	N/D	methanol:water (1:1, *v*/*v*)	[26]
	*S. hispanica*	N/D	methanol:water (1:1, *v*/*v*)	[26]
	*S. trachysperma*	N/D	methanol:water (1:1, *v*/*v*)	[26]
Diosmetin	*S. divaricata*	1.7 μg/g	methanol	[34]
Kaempferol	*S. hirsuta*	N/D	ethanol	[44]
	*S. laciniata*	3.55 ± 0.78 μg/g	methanol	[22]
	*S. latifolia*	0.62 ± 0.11 μg/g	methanol	[22]
Luteolin	*S. crispatula*	N/D	methanol:water (1:1, *v*/*v*)	[26]
	*S. divaricata*	0.21 μg/g	methanol	[34]
	*S. graminifolia*	N/D	ethanol	[44]
	*S. hirsuta*	N/D	ethanol	[44]
	*S. laciniata*	N/D	ethanol	[44]
	*S. mollis*	N/D	ethanol	[44]
	*S. pseudodivaricata*	N/D	methanol	[14]
	*S. pussila*	N/D	ethanol	[44]
	*S. trachysperma*	N/D	methanol:water (1:1, *v*/*v*)	[26]
	*S. villosa*	N/D	methanol:water (1:1, *v*/*v*)	[26]
2-(2,4-dihydroxyphenyl)-3,5,7-trihydroxychromen-4-one (morin)	*S. laciniata*	0.17 ± 0.01 μg/g	methanol	[22]
	*S. latifolia*	0.23 ± 0.04 μg/g	methanol	[22]
	*S. suberosa*	0.91 ± 0.83 μg/g	methanol	[22]
Myricetin	*S. laciniata*	4.45 ± 0.9 μg/g	methanol	[22]
	*S. latifolia*	16.16 ± 0.92 μg/g	methanol	[22]
	*S. suberosa*	3.12 ± 1.02 μg/g	methanol	[22]
Quercetin	*S. austriaca* var. *angustifolia*	N/D	ethanol	[44]
	*S. crispatula*	N/D	methanol:water (1:1, *v*/*v*)	[26]
	*S. graminifolia*	N/D	ethanol	[44]
	*S. hirsuta*	N/D	ethanol	[44]
	*S. hispanica*	N/D	methanol:water (1:1, *v*/*v*)	[26]
	*S. laciniata*	0.17 ± 0.01 μg/g	methanol	[22]
	*S. latifolia*	0.65 ± 0.15 μg/g	methanol	[22]
	*S. mollis*	N/D	ethanol	[44]
	*S. pussila*	N/D	ethanol	[44]
	*S. suberosa*	6.54 ± 1.16 μg/g	methanol	[22]
Quercetin derivative	*S. aristata*	N/D	methanol:water (1:1, *v*/*v*)	[26]
	*S. austriaca*	N/D	methanol:water (1:1, *v*/*v*)	[26]
Tricin	*S. divaricata*	0.16 μg/g	methanol	[34]
Unknown flavonoid	*S. hispanica*	N/D	methanol:water (1:1, *v*/*v*)	[26]
	*S. trachysperma*	N/D	methanol:water (1:1, *v*/*v*)	[26]
	*S. villosa*	N/D	methanol:water (1:1, *v*/*v*)	[26]
**Flavonoid *C*-glycosides**				
3′-methoxy-5,7,4′-trihydroxyflavone 6-*C*-β-D-glucopyranoside	*S. austriaca*	10 μg/g	70% aqueous ethanol	[24]
5,7,3′,4′-tetrahydroxyflavone 8-C-(6″-*O*-*trans*-caffeoyl β-D-glucopyranoside)	*S. austriaca*	100 μg/g	70% aqueous ethanol	[24]
5,7,3′,4′-tetrahydroxyflavone 6-C-(2″-*O*-β-D-glucopyranosyl β-D-glucopyranoside)	*S. austriaca*	15 μg/g	70%aqueous ethanol	[24]
5,7,4′-trihydroxyflavone 6-*C*-(2″-*O*-β-D-glucopyranosyl β-D-glucopyranoside)	*S. austriaca*	15 μg/g	70% aqueous ethanol	[24]
5,7,4′-trihydroxyflavone 6-*C*-β-D-glucopyranoside	*S. austriaca*	15 μg/g	70% aqueous ethanol	[24]
5,7,4′-trihydroxyflavone 8-*C*-(6″-*O*-*trans*-caffeoyl β-D-glucopyranoside)	*S. austriaca*	30 μg/g	70% aqueous ethanol	[24]
5,7-dihydroxy-2-(4-hydroxyphenyl)-6-[(2*S*,3*R*,4*R*,5*S*,6*R*)-3,4,5-trihydroxy-6-(hydroxymethyl)oxan-2-yl]-8-[(2*S*,3*R*,4*R*,5*R*,6*S*)-3,4,5-trihydroxy-6-methyloxan-2-yl]chromen-4-one (violanthin)	*S. radiata*	N/D	methanol	[13]
7-methylisoorientin	*S. latifolia*	6.4 μg/g	methanol	[4]
7-*O*-methylapigenin 6-*C*-β-*D*-glucopyranoside (swertisin)	*S. latifolia*	N/D	20% aqueous methanol	[10]
	*S. tomentosa*	N/D	20% aqueous methanol	[10]
Apigenin 3-C-α-L6-rhamnopyranosyl-8-C-β-D-glucopyranoside (scorzonerin B)	*S. radiata*	7.334 μg/g	methanol	[13]
Apigenin 6-C-glucoside (isovitexin)	*S. baetica*	N/D	methanol:water (1:1, *v*/*v*)	[26]
	*S. crispatula*	N/D	methanol:water (1:1, *v*/*v*)	[26]
Apigenin 6-*C*-β-D-galactopyranosyl-8-*C*-α-L-6-rhamnopyranoside (scorzonerin A)	*S. radiata*	30.667 μg/g	methanol	[13]
Apigenin 8-*C*-glucoside (vitexin)	*S. baetica*	N/D	methanol:water (1:1, *v*/*v*)	[26]
	*S. villosa*	N/D	methanol:water (1:1, *v*/*v*)	[26]
Apigenin di-*C*-glycoside (*C*-pentoside, *C*-hexoside)	*S. crispatula*	N/D	methanol:water (1:1, *v*/*v*)	[26]
	*S. trachysperma*	N/D	methanol:water (1:1, *v*/*v*)	[26]
Apigenin di-*C*-glycoside (di-*C*-hexoside)	*S. austriaca*	N/D	methanol:water (1:1, *v*/*v*)	[26]
	*S. baetica*	N/D	methanol:water (1:1, *v*/*v*)	[26]
	*S. crispatula*	N/D	methanol:water (1:1, *v*/*v*)	[26]
	*S. trachysperma*	N/D	methanol:water (1:1, *v*/*v*)	[26]
	*S. villosa*	N/D	methanol:water (1:1, *v*/*v*)	[26]
5,7-dihydroxy-2-(4-hydroxyphenyl)-8-[(2*S*,3*R*,4*R*,5*S*,6*R*)-3,4,5-trihydroxy-6-(hydroxymethyl)oxan-2-yl]-6-[(2*S*,3*R*,4*S*,5*S*)-3,4,5-trihydroxyoxan-2-yl]chromen-4-one (isoschaftoside)	*S. papposa*	3.334 μg/g	methanol	[62]
Luteolin 6-*C*-glucoside (isoorientin)	*S. aristata*	24.815 μg/g	methanol methanol:acetone:water (3/1/1, *v*/*v*/*v*)	[56]
	*S. austriaca*	N/D	methanol:water (1:1, *v*/*v*)	[26]
	*S. baetica*	N/D	methanol:water (1:1, *v*/*v*)	[26]
	*S. crispatula*	N/D	methanol:water (1:1, *v*/*v*)	[26]
	*S. hispanica*	N/D	methanol:water (1:1, *v*/*v*)	[26]
	*S. latifolia*	9 μg/g	methanol	[4]
	*S. papposa*	48.667 μg/g	methanol	[62]
	*S. radiata*	N/D	methanol	[13]
	*S. trachysperma*	N/D	methanol:water (1:1, *v*/*v*)	[26]
	*S. villosa*	N/D	methanol:water (1:1, *v*/*v*)	[26]
Luteolin 8-*C*-glucoside (orientin)	*S. aristata*	N/D	methanol:water (1:1, *v*/*v*)	[26]
	*S. austriaca*	N/D	methanol:water (1:1, *v*/*v*)	[26]
	*S. baetica*	N/D	methanol:water (1:1, *v*/*v*)	[26]
	*S. papposa*	6.667 μg/g	methanol	[62]
Luteolin di-*C*-glycoside (*C*-hexoside, *C*-pentoside)	*S. crispatula*	N/D	methanol:water (1:1, *v*/*v*)	[26]
	*S. hispanica*	N/D	methanol:water (1:1, *v*/*v*)	[26]
	*S. trachysperma*	N/D	methanol:water (1:1, *v*/*v*)	[26]
Swertiajaponin	*S. papposa*	3.667 μg/g	methanol	[62]
**Flavonoid *O*-glycosides and *O*-glucuronides**				
Apigenin 7-*O*-glucoside (apigetrin)	*S. villosa*	N/D	methanol:water (1:1, *v*/*v*)	[26]
Apigenin 7-*O*-glucuronide	*S. trachysperma*	N/D	methanol:water (1:1, *v*/*v*)	[26]
	*S. villosa*	N/D	methanol:water (1:1, *v*/*v*)	[26]
Kaempferol 3-*O*-rutinoside	*S. divaricata*	N/D	methanol	[14]
	*S. radiata*	N/D	methanol	[13]
Luteolin 5-*O*-glucoside	*S. pseudodivaricata*	N/D	methanol	[14]
Luteolin 7-*O*-glucoside (cynaroside)	*S. acuminata*	9.583 ± 0.203 μg/mg	20% aqueous methanol	[9]
	*S. baetica,*	N/D	methanol:water (1:1, *v*/*v*)	[26]
	*S. cinerea*	81.14 ± 0.62 μg/mg	20% aqueous methanol	[5]
	*S. incisa*	12.08 ± 0.1 μg/mg	20% aqueous methanol	[5]
	*S. latifolia*	629.23 ± 3.53 μg/mg	20% aqueous methanol	[5]
	*S. mollis* ssp. *szowitsii*	107.43 ± 0.09 μg/mg	20% aqueous methanol	[5]
	*S. parviflora*	51.80 ± 0.71 μg/mg	20% aqueous methanol	[5]
	*S. pseudodivaricata*	N/D	methanol	[14]
	*S. tomentosa*	47.81 ± 0.50 μg/mg	20% aqueous methanol	[5]
	*S. villosa*	N/D	methanol:water (1:1, *v*/*v*)	[26]
Luteolin 7-*O*-glucuronide	*S. trachysperma*	N/D	methanol:water (1:1, *v*/*v*)	[26]
	*S. villosa*	N/D	methanol:water (1:1, *v*/*v*)	
Quercetin 3-*O*-arabinofuranoside (avicularin)	*S. austriaca*	N/D	methanol:water (1:1, *v*/*v*)	[26]
Quercetin 3-*O*-galactoside (hyperoside)	*S. cinerea*	124.22 ± 0.56 μg/mg	20% aqueous methanol	[5]
	*S. hispanica*	11.41 ± 0.05 mg/g 11.35 ± 0.15 mg/g	methanol:acetone:water (3:1:1)	[20]
	*S. latifolia*	305.71 ± 1.70 μg/mg	20% aqueous methanol	[5]
	*S. mollis* ssp. *szowitsii*	39.46 ± 0.03 μg/mg	20% aqueous methanol	[5]
	*S. parviflora*	9.71 ± 0.51 μg/mg	20% aqueous methanol	[5]
	*S. tomentosa*	94.54 ± 0.33 μg/mg	20% aqueous methanol	[5]
	*S. villosa*	N/D	methanol:water (1:1, *v*/*v*)	[26]
Quercetin 3-*O*-glucoside (isoquercitrin)	*S. aristata*	40.37 μg/g	methanol methanol:acetone:water (3/1/1, *v*/*v*/*v*)	[56]
	*S. austriaca*	N/D	methanol:water (1:1, *v*/*v*)	[26]
	*S. hispanica*	6.41 ± 0.02 mg/g 6.91 ± 0.01 mg/g	methanol:acetone:water (3/1/1, *v*/*v*/*v*)	[20]
	*S. villosa*	N/D	methanol:water (1:1, *v*/*v*)	[26]
Quercetin 3-*O*-glucuronide (miquelianin)	*S. hispanica*	N/D	methanol:water (1:1, *v*/*v*)	[26]
		13.87 ± 0.10 mg/g 15.29 ± 0.25 mg/g	methanol:acetone:water (3/1/1, *v*/*v*/*v*)	[20]
Quercetin 3-*O*-rhamnoglucoside (rutin)	*S. acuminata*	597.335 ± 1.104 μg/mg	20% aqueous methanol	[9]
	*S. aristata*	36.667 μg/g	methanol methanol:acetone:water (3/1/1, *v*/*v*/*v*)	[56]
	*S. austriaca*	N/D	methanol:water (1:1, *v*/*v*)	[26]
	*S. incisa*	198.81 ± 0.18 μg/mg	20% aqueous methanol	[5]
	*S. mollis* ssp. *szowitsii*	26.32 ± 0.04 μg/mg	20% aqueous methanol	[5]
	*S. radiata*	N/D	methanol	[13]
	*S. suberosa*	15.38 ± 3.27 μg/g	methanol	[22]
Quercetin 3-*O*-α-rhamnopyranosyl-(1→6)-β-D-galactopyranoside	*S. latifolia*	2.267 μg/g	methanol	[4]
Quercetin 3-*O*-β-apiofuranosyl-(1‴→2″)-β-D-glucopyranoside	*S. latifolia*	11.53 μg/g	methanol	[4]
Quercetin 3-*O*-β-D-glucoside	*S. latifolia*	N/D	20% aqueous methanol	[10]
	*S. tomentosa*	N/D	20% aqueous methanol	
Quercetin *O*-mallonylhexoside	*S. aristata*	N/D	methanol:water (1:1, *v*/*v*)	[26]
	*S. hispanica*	2.65 ± 0.05 μg/mg 2.83 ± 0.01 μg/mg	methanol:acetone:water (3/1/1, *v*/*v*/*v*)	[20]
**Flavonoid *O*-*C*-glycosides**				
Apigenin *O*-*C*-glycoside (*O*-hexoside, *C*-hexoside)	*S. trachysperma*	N/D	methanol:water (1:1, *v*/*v*)	[26]
Apigenin O-C-glycoside (*O*-pentoside, *C*-hexoside)	*S. crispatula*	N/D	methanol:water (1:1, *v*/*v*)	[26]
	*S. trachysperma*	N/D	methanol:water (1:1, *v*/*v*)	[26]
Isovitexin 2′-*O*-xyloside	*S. divaricata*	N/D	methanol	[14]
Isovitexin 2″-*O*-xyloside	*S. pseudodivaricata*	N/D	methanol	[14]
Isovitexin 4′-*O*-glucoside	*S. divaricata*	N/D	methanol	[14]
Luteolin *O*-*C*-glycoside (*O*-hexoside, *C*-hexoside)	*S. austriaca*	N/D	methanol:water (1:1, *v*/*v*)	[26]
	*S. baetica*	N/D	methanol:water (1:1, *v*/*v*)	[26]
	*S. crispatula*	N/D	methanol:water (1:1, *v*/*v*)	[26]
	*S. trachysperma*	N/D	methanol:water (1:1, *v*/*v*)	[26]
Luteolin *O*-*C*-glycoside (*O*-pentoside, *C*-hexoside)	*S. aristata*	N/D	methanol:water (1:1, *v*/*v*)	[26]
	*S. austriaca*	N/D	methanol:water (1:1, *v*/*v*)	[26]
	*S. crispatula*	N/D	methanol:water (1:1, *v*/*v*)	[26]
	*S. trachysperma*	N/D	methanol:water (1:1, *v*/*v*)	[26]
**Macrolides**				
Sacrolide A	*S. divaricata*	0.6 μg/g	methanol	[34]
**Organic acids/Phenolic acids and their derivatives**				
1,5-*O*-dicaffeoylquinic acid	*S. baetica*	N/D	methanol:water (1:1, *v*/*v*)	[26]
	*S. crispatula*	N/D	methanol:water (1:1, *v*/*v*)	[26]
	*S. hispanica*	3.95 ± 0.11 μg/mg 6.59 ± 0.17 μg/mg	methanol:acetone:water (3/1/1, *v*/*v*/*v*)	[20]
	*S. trachysperma*	N/D	methanol:water (1:1, *v*/*v*)	[26]
	*S. villosa*	N/D	methanol:water (1:1, *v*/*v*)	[26]
3,4-bis[(3′,4-dioxo-1′,3′,5′,6′-tetrahydrospiro[cyclohexa-2,5-diene-1,4′-cyclopenta[c]-furan]-1′-yl)]chlorogenic acid	*S. aucheriana*	7.443 μg/g	methanol	[29]
3,4-*O*-dihydroxyphenyl caffeate	*S. aucheriana*	7.323 μg/g	methanol	[7]
3,5-*O*-dicaffeoyl quinic acid	*S. pseudodivaricata*	N/D	methanol	[14]
3,5-*O*-dicaffeoyl-*epi*-quinic acid	*S. radiata*	N/D	methanol	[12,13]
	*S. aucheriana*	6.243 μg/g	methanol	[7]
3,5-*O*-dicaffeoylquinic acid methyl ester (macroantoin G)	*S. radiata*	N/D	methanol	[13]
3,5-*O*-dicaffeoylquinic acid (isochlorogenic acid A)	*S. aristata*	23.334 μg/g	methanol methanol:acetone:water (3/1/1, *v*/*v*/*v*)	[56]
	*S. austriaca*	N/D	methanol:water (1:1, *v*/*v*)	[26]
	*S. baetica*	N/D	methanol:water (1:1, *v*/*v*)	[26]
	*S. crispatula*	N/D	methanol:water (1:1, *v*/*v*)	[26]
	*S. hispanica*	35.15 ± 0.61 μg/mg 19.23 ± 0.58 μg/mg	methanol:acetone:water (3/1/1, *v*/*v*/*v*)	[20]
	*S. radiata*	N/D	methanol	[13]
	*S. trachysperma*	N/D	methanol:water (1:1, *v*/*v*)	[26]
	*S. villosa*	N/D	methanol:water (1:1, *v*/*v*)	[26]
3-*O*-caffeoylquinic acid (chlorogenic acid)	*S. acuminata*	372.128 ± 0.961 μg/mg	20% aqueous methanol	[9]
	*S. aristata*	9.259 μg/g	methanol methanol:acetone:water (3/1/1, *v*/*v*/*v*)	[56]
	*S. austriaca*	N/D	methanol:water (1:1, *v*/*v*)	[26]
	*S. baetica*	N/D	methanol:water (1:1, *v*/*v*)	[26]
	*S. cinerea*	266.51 ± 1.51 μg/mg	20% aqueous methanol	[5]
	*S. crispatula*	N/D	methanol:water (1:1, *v*/*v*)	[26]
	*S. divaricata*	N/D	methanol	[14]
	*S. hispanica*	85.49 ± 1.49 μg/mg 75.83 ± 1.01 μg/mg	methanol:acetone:water (3/1/1, *v*/*v*/*v*)	[20]
	*S. incisa*	569.19 ± 1.62 μg/mg	20% aqueous methanol	[5]
	*S. latifolia*	652.32 ± 2.48 μg/mg	20% aqueous methanol	[5]
	*S. mollis* ssp. *szowitsii*	1032.16 ± 2.05 μg/mg	20% aqueous methanol	[5]
	*S. parviflora*	444.77 ± 2.78 μg/mg	20% aqueous methanol	[5]
	*S. radiata*	N/D	methanol	[13]
	*S. tomentosa*	268.75 ± 1.72 μg/mg	20% aqueous methanol	[5]
	*S. trachysperma*	N/D	methanol:water (1:1, *v*/*v*)	[26]
	*S. villosa*	N/D	methanol:water (1:1, *v*/*v*)	[26]
3-*O*-feruloyl-1,4-di-O-dihydrocaffeoylquinic acid (feruloylpodospermic acid B)	*S. divaricata*	23.438 μg/g	methanol	[14]
3-*O*-feruloyl-1,5-di-*O*-dihydrocaffeoylquinic acid (feruloylpodospermic acid A)	*S. divaricata*	82.031 μg/g	methanol	[14]
4,5-dicaffeoyl-*epi*-quinic acid	*S. radiata*	10.333 μg/g	methanol	[13]
4,5-dicaffeoyl-*epi*-quinic acid methyl ester (macroantoin F)	*S. radiata*	N/D	methanol	[13]
4,5-*O*-dicaffeoylquinic acid (isochlorogenic acid C)	*S. aristata*	13.33 μg/g	methanol methanol:acetone:water (3/1/1, *v*/*v*/*v*)	[56]
	*S. austriaca*	N/D	methanol:water (1:1, *v*/*v*)	[26]
	*S. baetica*	N/D	methanol:water (1:1, *v*/*v*)	[26]
	*S. crispatula*	N/D	methanol:water (1:1, *v*/*v*)	[26]
	*S. hispanica*	5.42 ± 0.01 μg/mg 3.14 ± 0.15 μg/mg	methanol:acetone:water (3/1/1, *v*/*v*/*v*)	[20]
	*S. radiata*	N/D	methanol	[13]
	*S. trachysperma*	N/D	methanol:water (1:1, *v*/*v*)	[26]
	*S. villosa*	N/D	methanol:water (1:1, *v*/*v*)	[26]
4-hydroxy-3-methoxyphenyl ferulate	*S. divaricata*	0.13 μg/g	methanol	[34]
4-*O*-caffeoylquinic acid (cryptochlorogenic acid)	*S. aristata*	N/D	methanol:water (1:1, *v*/*v*)	[26]
	*S. austriaca*	N/D	methanol:water (1:1, *v*/*v*)	[26]
	*S. baetica*	N/D	methanol:water (1:1, *v*/*v*)	[26]
	*S. crispatula*	N/D	methanol:water (1:1, *v*/*v*)	[26]
	*S. hispanica*	2.99 ± 0.03 μg/mg 3.99 ± 0.04 μg/mg	methanol:acetone:water (3/1/1, *v*/*v*/*v*)	[20]
	*S. trachysperma*	N/D	methanol:water (1:1, *v*/*v*)	[26]
	*S. villosa*	N/D	methanol:water (1:1, *v*/*v*)	[26]
5-*O*-caffeoylquinic acid (*cis*-chlorogenic acid)	*S. baetica*	N/D	methanol:water (1:1, *v*/*v*)	[26]
	*S. crispatula*	N/D	methanol:water (1:1, *v*/*v*)	[26]
	*S. hispanica*	N/D	methanol:water (1:1, *v*/*v*)	[26]
	*S. trachysperma*	N/D	methanol:water (1:1, *v*/*v*)	[26]
	*S. villosa*	N/D	methanol:water (1:1, *v*/*v*)	[26]
5-*p*-coumaroylquinic acid (*cis*)	*S. radiata*	N/D	methanol	[12]
5-*p*-coumaroylquinic acid (*trans*)	*S. radiata*	N/D	methanol	[12,13]
Methyl 1-(2-methylcyclopropyl-1-carbonyloxy)chlorogenate	*S. aucheriana*	7.683 μg/g	methanol	[29]
Quinic acid	*S. radiata*	N/D	methanol	[12,13]
*Trans*-caffeic acid	*S. divaricata*	1.8 μg/g	methanol	[34]
*Trans*-*p*-hydroxy coumaric acid	*S. divaricata*	1 μg/g	methanol	[34]
**Sesquiterpenoids**				
1β,4α- dihydroxy-5α,6β,7α,11βH-eudermn-12,6-olide	*S. divaricata*	0.038 μg/g	methanol	[34]
5-(1-(2-*O*-hexanoyl)-β-D-glucopyranosyloxy)-2-hydroxy-3-[4-(4-hydroxyphenyl)-2-oxobutyl]benzoic acid (scorzoneric acid)	*S. pseudodivaricata*	14.5 μg/g	methanol	[14]
8*R*-matricarinyl 3-[4-(1-β-D-glucopyranosyloxy)phenyl]propanoate (scorzonerin)	*S. pseudodivaricata*	260 μg/g	methanol	[14]
Glucozaluzanin C	*S. divaricata*	1.56 μg/g	methanol	[34]
Sulfoscorzonin D	*S. divaricata*	1.3 μg/g	methanol	[34]
Sulfoscorzonin E	*S. divaricata*	1.25 μg/g	methanol	[34]
**Steroids**				
(22*E*)-5α,8α-epidioxyergosta-6,22-dien-3β-ol	*S. divaricata*	0.76 μg/g	methanol	[34]
Ergosta-3β,5α,6β-trialcohol	*S. divaricata*	0.7 μg/g	methanol	[34]
Ergosterol	*S. laciniata*	2.44 ± 0.11 μg/g	hexane/isopropanol (3:2, *v*/*v*)	[22]
	*S. latifolia*	3.22 ± 0.09 μg/g	hexane/isopropanol (3:2, *v*/*v*)	[22]
	*S. suberosa*	3.06 ± 0.41 μg/g	hexane/isopropanol (3:2, *v*/*v*)	[22]
Stigma-5-en-3-*O*-β-glucoside	*S. divaricata*	3.5 μg/g	methanol	[34]
Stigmasterol	*S. laciniata*	21.67 ± 1.1 μg/g	hexane/isopropanol (3:2, *v*/*v*)	[22]
	*S. latifolia*	30.76 ± 1.19 μg/g	hexane/isopropanol (3:2, *v*/*v*)	[22]
	*S. suberosa*	10.80 ± 0.54 μg/g	hexane/isopropanol (3:2, *v*/*v*)	[22]
β-Sitosterol	*S. aucheriana*	11.765 μg/g	methanol	[29]
	*S. laciniata*	4.26 ± 0.34 μg/g	hexane/isopropanol (3:2, *v*/*v*)	[22]
	*S. latifolia*	35.55 ± 1.71 μg/g	hexane/isopropanol (3:2, *v*/*v*)	[22]
	*S. suberosa*	50.75 ± 3.15 μg/g	hexane/isopropanol (3:2, *v*/*v*)	[22]
**Triterpenoids**				
3β-hydroxy-fern-7-en-6-one-acetate	*S. latifolia*	18 ± 1 μg/g	*n*-hexane	[27]
Lup-20(29)-ene3β,28-diol	*S. divaricata*	3 μg/g	methanol	[34]
Lupeol	*S. acuminata*	327 ± 5 μg/g	*n*-hexane	[27]
	*S. aucheriana*	9.724 μg/g	methanol	[29]
	*S. cana* var. *jacquiniana*	932 ± 2 μg/g	*n*-hexane	[27]
	*S. cinerea*	1174 ± 16 μg/g	*n*-hexane	[27]
	*S. eriophora*	228 ± 6 μg/g	*n*-hexane	[27]
	*S. incisa*	1090 ± 2 μg/g	*n*-hexane	[27]
	*S. laciniata* ssp. *laciniata*	932 ± 2 μg/g	*n*-hexane	[27]
	*S. latifolia*	1538 ± 1 μg/g	*n*-hexane	[27]
	*S. mirabilis*	954 ± 14 μg/g	*n*-hexane	[27]
	*S. mollis* ssp. *szowitsii*	321 ± 1 μg/g	*n*-hexane	[27]
	*S. parviflora*	649 ± 6 μg/g	*n*-hexane	[27]
	*S. suberosa* ssp. *suberosa*	1005 ± 17 μg/g	*n*-hexane	[27]
	*S. sublanata*	169 ± 1 μg/g	*n*-hexane	[27]
	*S. tomentosa*	509 ± 2 μg/g	*n*-hexane	[27]
Lupeol acetate	*S. acuminata*	67 ± 1 μg/g	*n*-hexane	[27]
	*S. aucheriana*	5.642 μg/g	methanol	[29]
	*S. cana* var. *jacquiniana*	535 ± 4 μg/g	*n*-hexane	[27]
	*S. cinerea*	839 ± 6 μg/g	*n*-hexane	[27]
	*S. eriophora*	368 ± 1 μg/g	*n*-hexane	[27]
	*S. incisa*	236 ± 9 μg/g	*n*-hexane	[27]
	*S. laciniata* ssp. *laciniata*	892 ± 2 μg/g	*n*-hexane	[27]
	*S. latifolia*	607 ± 1 μg/g	*n*-hexane	[27]
	*S. mirabilis*	998 ± 13 μg/g	*n*-hexane	[27]
	*S. mollis* ssp. *szowitsii*	149 ± 7 μg/g	*n*-hexane	[27]
	*S. parviflora*	594 ± 5 μg/g	*n*-hexane	[27]
	*S. suberosa* ssp. *suberosa*	312 ± 4 μg/g	*n*-hexane	[27]
	*S. sublanata*	302 ± 1 μg/g	*n*-hexane	[27]
	*S. tomentosa*	411 ± 1 μg/g	*n*-hexane	[27]
Oleanolic acid	*S. divaricata*	1.5 μg/g	methanol	[34]
Ptiloepoxide	*S. aucheriana*	14.646 μg/g	methanol	[29]
Taraxasterol	*S. aucheriana*	30.972 μg/g	methanol	[29]
Taraxasteryl acetate/Taraxasterol acetate	*S. aucheriana*	5.552 μg/g	methanol	[29]
	*S. cana* var. *jacquiniana*	81 ± 3 μg/g	*n*-hexane	[9]
	*S. cinerea*	417 ± 11 μg/g	*n*-hexane	[27]
	*S. eriophora*	545 ± 5 μg/g	*n*-hexane	[27]
	*S. incisa*	280 ± 10 μg/g	*n*-hexane	[27]
	*S. laciniata* ssp. *laciniata*	69 ± 5 μg/g	*n*-hexane	[27]
	*S. latifolia*	1062 ± 2 μg/g	*n*-hexane	[27]
	*S. mirabilis*	1262 ± 728 μg/g	*n*-hexane	[27]
	*S. mollis* ssp. *szowitsii*	263 ± 4 μg/g	*n*-hexane	[27]
	*S. parviflora*	433 ± 2 μg/g	*n*-hexane	[27]
	*S. suberosa* ssp. *suberosa*	535 ± 4 μg/g	*n*-hexane	[27]
	*S. sublanata*	4981 ± 2 μg/g	*n*-hexane	[27]
	*S. tomentosa*	376 ± 13 μg/g	*n*-hexane	[27]
Taraxasterol oleate	*S. aucheriana*	36.255 μg/g	methanol	[29]
α-Amyrin	*S. acuminata*	1102 ± 6 μg/g	*n*-hexane	[27]
	*S. cana* var. *jacquiniana*	442 ± 5 μg/g	*n*-hexane	[27]
	*S. cinerea*	309 ± 2 μg/g	*n*-hexane	[27]
	*S. incisa*	644 ± 2 μg/g	*n*-hexane	[27]
	*S. laciniata* ssp. *laciniata*	209 ± 3 μg/g	*n*-hexane	[27]
	*S. latifolia*	827 ± 2 μg/g	*n*-hexane	[27]
	*S. mollis* ssp. *szowitsii*	246 ± 8 μg/g	*n*-hexane	[27]
**Vomifoliols**				
Vomifoliol	*S. divaricata*	0.7 μg/g	methanol	[34]

Compound concentration was taken directly from literature or it was calculated, dividing the mass of the isolated compound by the mass of plant material used for extraction; N/D—no data was available in the literature.

**Table 2:** Compounds present in subaerial parts of *Scorzonera* species.

Compounds	*Scorzonera* Species	Concentration	Type of Solvent	References
**Coumarins and coumarin derivatives**				
Scopoletin	*S. divaricata*	2.546 μg/g	95% aqueous ethanol	[57]
Scopolin	*S. divaricata*	16.364 μg/g	95% aqueous ethanol	[57]
Coumarin *O*-β-glycoside (cichoriin)	*S. undulata* ssp. *deliciosa*	12.99 μg/g	dichloromethane	[63]
	*S. cana* var. *jacquiniana*	6.667 μg/g	methanol	[72]
Scorzonerol	*S. pygmaea*	0.857 μg/g	ethanol	[35]
**Dihydroisocoumarins and dihydroisocoumarin glycosides**				
(‒)-Hydrangenol 4′-*O*-glucoside	*S. tomentosa*	20.101 μg/g	methanol	[36]
	*S. judaica*	14.286 μg/g	chloroform:methanol (9:1) methanol	[25]
(‒)-Scorzotomentosin 4′-*O*-β-glucoside	*S. tomentosa*	158.29 μg/g	methanol	[36]
	*S. latifolia*	N/D	methanol:water (8:2)	[73]
(±)-Hydrangenol	*S. tomentosa*	82.915 μg/g	methanol	[36]
	*S. judaica*	51.143 μg/g	chloroform chloroform:methanol (9:1)	[25]
(3RS)-3,4-dihydro-3-(4-hydroxyphenyl)-8-methoxy-1*H*-2-benzopyran-1-one ((±)-scorzotomentosin)	*S. tomentosa*	216.08 μg/g	methanol	[36]
	*S. judaica*	8.714 μg/g	chloroform	[25]
8-*O*-[α-L-rhamnopyranosyl(1→6)-β-D-glucopyranosyl]scorzocreticin (scorzocreticoside II)	*S. pygmaea*	21.429 μg/g	ethanol	[35]
8-*O*-β-D-glucopyranosylscorzocreticin (scorzocreticoside I)	*S. pygmaea*	14.286 μg/g	ethanol	[35]
Hydrangenol 4′-*O*-β-D-apiofuranosyl-(1→6)-β-D-glucopyranoside	*S. judaica*	22.143 μg/g	chloroform:methanol (9:1) methanol	[25]
3S-hydrangenol 4′-*O*-α-L-rhamnopyranosyl-(1→3)-β-D-glucopyranoside	*S. judaica*	48.571 μg/g	chloroform:methanol (9:1) methanol	[25]
Hydrangenol 8-*O*-β-D-glucopyranoside	*S. judaica*	14.143 μg/g	chloroform:methanol (9:1) methanol	[25]
Scorzopygmaecoside	*S. pygmaea*	14.286 μg/g	ethanol	[35]
Thunberginol C	*S. pygmaea*	4.714 μg/g	ethanol	[35]
Thunberginol F	*S. judaica*	7.429 μg/g	chloroform:methanol (9:1)	[25]
Thunberginol G	*S. papposa*	38.4 μg/g	methanol	[62]
**Fatty acids**				
Linoleic acid	*S. divaricata*	1.091 μg/g	95% aqueous ethanol	[57]
	*S. hispanica*	7.143 μg/g	ethyl acetate	[20]
9*S*,12*S*,13*S*-trihydroxy-10*E*-octadecenoate	*S. divaricata*	0.546 μg/g	95% aqueous ethanol	[57]
Palmitic acid	*S. divaricata*	0.727 μg/g	95% aqueous ethanol	[57]
**Flavonoid aglycones**				
Galangustin	*S. undulata* ssp. *deliciosa*	15.464 μg/g	dichloromethane	[49,63]
**Flavonoid *C*-glycosides**				
Luteolin 6-*C*-glucoside (isoorientin)	*S. cana* var. *jacquiniana*	21.667 μg/g	methanol	[72]
Luteolin 8-*C*-glucoside (orientin)	*S. cana* var. *jacquiniana*	20 μg/g	methanol	[72]
Apigenin 8-*C*-glucoside (vitexin)	*S. cana* var. *jacquiniana*	7.133 μg/g	methanol	[72]
**Flavonoid O-glycosides**				
Apigenin 7-*O*-β-glucoside	*S. cana* var. *jacquiniana*	18.333 μg/g	methanol	[72]
Luteolin 7-*O*-β-glucoside	*S. cana* var. *jacquiniana*	12.55 μg/g	methanol	[72]
Apigenin 7-*O*-β-rutinoside	*S. cana* var. *jacquiniana*	25 μg/g	methanol	[72]
**Hydroquinone derivatives**				
Arbutin	*S. cana* var. *jacquiniana*	13.333 μg/g	methanol	[72]
6′-*O*-caffeoylarbutin	*S. cana* var. *jacquiniana*	10.5 μg/g	methanol	[72]
**Lignans**				
Pinoresinol-1-yl-β-D-glucopyranoside	*S. humilis*	90.147 μg/g	methanol	[54]
(‒)-Syringaresinol	*S. hispanica*	9.643 μg/g	ethyl acetate	[20]
Pinoresinol	*S. divaricata*	0.909 μg/g	95% aqueous ethanol	[57]
4-[β-D-glucopyranosyl)hydroxy]-pinoresinol (pinoresinol-4-*O*-glucoside)	*S. judaica*	15.857 μg/g	chloroform:methanol (9:1) methanol	[25]
4α-hydroxypinoresinol	*S. judaica*	26.429 μg/g	chloroform chloroform:methanol (9:1)	[25]
**Organic acids/Phenolic acids and their derivatives**				
(–)–1,4-di-*O*-feruloyl-3-*O*-dihydrocaffeoylquinic acid	*S. divaricata*	5.363 μg/g	95% aqueous ethanol	[58]
(–)–1-*O*-feruloyl-3-*O*-dihydrocaffeoylquinic acid	*S. divaricata*	N/D	95% aqueous ethanol	[58]
(–)–1-*O*-feruloyl-4-*O*-dihydrocaffeoylquinic acid	*S. divaricata*	0.909 μg/g	95% aqueous ethanol	[58]
(–)–1-*O*-feruloyl-5-*O*-dihydrocaffeoylquinic acid	*S. divaricata*	N/D	95% aqueous ethanol	[58]
(–)–3,5-di-*O*-feruloylquinic acid	*S. divaricata*	1.818 μg/g	95% aqueous ethanol	[58]
1,3-di-*O*-caffeoylquinic acid methyl ester	*S. hieraciifolia*	N/D	ethanol	[8]
1,5-di-*O*-feruloylquinic acid	*S. hieraciifolia*	N/D	ethanol	[8]
1,5-*O*-dicaffeoylquinic acid	*S. hispanica*	2.26–12.72 μg/mg	methanol:acetone:water (3/1/1, *v*/*v*/*v*)	[20]
13-*Oxo*-(9*E*,11*E*)-octadecadienoic acid	*S. hispanica*	3.929 μg/g	ethyl acetate	[20]
13-*Oxo*-(9*Z*,11*E*)-octadecadienoic acid	*S. hispanica*	2.679 μg/g	ethyl acetate	[20]
2-Hydroxy-6-[2-(3,4-dihydroxyphenyl)-2-*oxo*-ethyl]benzoic acid	*S. judaica*	5 μg/g	methanol	[25]
2-Hydroxy-6-[2-(3,4-dihydroxyphenyl-5-methoxy)-2-oxoethyl]benzoic acid	*S. judaica*	3.714 μg/g	methanol	[25]
2-Hydroxy-6-[2-(4-hydroxyphenyl)-2-oxo-ethyl]benzoic acid	*S. judaica*	17.143 μg/g	chloroform:methanol (9:1) methanol	[25]
3-(4′-Hydroxyphenyl)-2-propenoic acid (4″-carboxyl)-phenyl ester	*S. hieraciifolia*	N/D	ethanol	[8]
3,5-di-*O*-caffeoylquinic acid	*S. aristata*	13.735 μg/g	methanol:acetone:water (3/1/1, *v*/*v*/*v*) methanol	[56]
	*S. hispanica*	1.04–52.13 μg/mg	methanol:acetone:water (3/1/1, *v*/*v*/*v*)	[20]
	*S. humilis*	N/D	methanol	[55]
	*S. latifolia*	12.5 μg/g	methanol	[67]
	*S. pygmaea*	3.214 μg/g	ethanol	[35]
3,5-dicaffeoylquinic acid methyl ester (macroantoin G)	*S. cana* var. *jacquiniana*	10 μg/g	methanol	[72]
	*S. hieraciifolia*	N/D	ethanol	[8]
3-*O*-caffeoylquinic acid methyl ester	*S. hieraciifolia*	N/D	ethanol	[8]
4,5-dicaffeoylquinic acid (isochlorogenic acid C)	*S. hispanica*	2.46–4.59 μg/mg	methanol:acetone:water (3/1/1, *v*/*v*/*v*)	[20]
	*S. latifolia*	2.5 μg/g	methanol	[67]
	*S. veratrifolia*	25.667 μg/g	methanol	[64]
4,5-di-*O*-caffeoylquinic acid methyl ester	*S. latifolia*	25 μg/g	methanol	[67]
	*S. hieraciifolia*	N/D	ethanol	[8]
4-Hydroxybenzoic acid 4-(6-*O*-α-rhamnopyranosyl-β-glucopyranosyl) benzyl ester	*S. cana* var. *jacquiniana*	20.433 μg/g	methanol	[72]
4-*O*-caffeoylquinic acid (cryptochlorogenic acid)	*S. hispanica*	0.52–0.93 μg/mg	methanol:acetone:water (3/1/1, *v*/*v*/*v*)	[20]
5-*O*-feruloyl quinic acid methyl ester	*S. hieraciifolia*	N/D	ethanol	[8]
9-Hydroxyocta-(10*E*,12*E*)-decadienoic acid	*S. hispanica*	N/D	ethyl acetate	[20]
9-*Oxo*-(10*E*,12*E*)-octadecadienoic acid	*S. hispanica*	1.071 μg/g	ethyl acetate	[20]
9-*Oxo*-(10*E*,12*Z*)-octadecadienoic acid	*S. hispanica*	N/D	ethyl acetate	[20]
Butyl 3-*O*-feruloylquinate	*S. divaricata*	2.909 μg/g	95% aqueous ethanol	[58]
Caffeic acid	*S. divaricata*	2.909 μg/g	95% aqueous ethanol	[57]
	*S. hieraciifolia*	N/D	ethanol	[8]
	*S. hispanica*	0.13–2.47 μg/g	methanol:acetone:water (3/1/1, *v*/*v*/*v*)	[20]
	*S. latifolia*	4.58 μg/g	Methanol	[67]
Caffeic acid methyl ester	*S. aristata*	56.627 μg/g	methanol:acetone:water (3/1/1, *v*/*v*/*v*) methanol	[56]
Chlorogenic acid	*S. cinerea*	412.89 ± 0.55 μg/mg	20% aqueous methanol	[5]
	*S. hispanica*	3.80–43.82 μg/mg	methanol:acetone:water (3/1/1, *v*/*v*/*v*)	[20]
	*S. humilis*	N.D	methanol	[55]
	*S. incisa*	141.49 ± 0.20 μg/mg	20% aqueous methanol	[5]
	*S. latifolia*	1246.78 ± 3.20 μg/mg	20% aqueous methanol	[5]
	*S. mollis* ssp. *szowitsii*	159.25 ± 0.24 μg/mg	20% aqueous methanol	[5]
	*S. parviflora*	509.96 ± 6.64 μg/mg	20% aqueous methanol	[5]
	*S. pygmaea*	3.43 μg/g	ethanol	[35]
	*S. tomentosa*	734.72 ± 1.04 μg/mg	20% aqueous methanol	[5]
	*S. veratrifolia*	61.857 μg/g	methanol	[64]
Chlorogenic acid methyl ester	*S. hieraciifolia*	N/D	ethanol	[8]
	*S. latifolia*	3.75 μg/g	methanol	[67]
	*S. pygmaea*	10 μg/g	ethanol	[35]
	*S. veratrifolia*	31.667 μg/g	methanol	[64]
Cryptochlorogenic acid	*S. veratrifolia*	11 μg/g	methanol	[64]
Dihydrocaffeic acid	*S. divaricata*	1.091 μg/g	95% aqueous ethanol	[57]
Dihydrocaffeic acid ethyl ester (ethyl dihydrocaffeate)	*S. divaricata*	1.091 μg/g	95% aqueous ethanol	[57]
Dihydrocaffeic acid methyl ester (methyl dihydrocaffeate)	*S. divaricata*	0.909 μg/g	95% aqueous ethanol	[57]
Dihydrocaffeic acid n-butyl ester (propyl dihydrocaffeate)	*S. divaricata*	7.273 μg/g	95% aqueous ethanol	[57]
*E*-3-(3,4-dihydroxybenzylidene)-5-(3,4-dihydroxyphenyl)dihydrofuran-2-one	*S. judaica*	9.714 μg/g	chloroform:methanol (9:1)	[25]
Hydrangeic acid 4′-*O*-β-D-glucopyranoside	*S. judaica*	3.857 μg/g	methanol	[25]
Methyl 3-O-feruloylquinate	*S. divaricata*	0.727 μg/g	95% aqueous ethanol	[58]
Protocatechuic acid	*S. cana* var. *jacquiniana*	8.5 μg/g	methanol	[72]
*Z*-3-(3,4-dihydroxybenzylidene)-5-(3,4-dihydroxyphenyl)-2(3*H*)-furanone	*S. judaica*	4.571 μg/g	chloroform:methanol (9:1)	[25]
**Phtalides**				
(3*RS*)-3-[(*SR*)-hydroxy(4-hydroxyphenyl)-methyl]-7-methoxy-2-benzofuran-1(3*H*)-one ((±)-scorzophthalide)	*S. tomentosa*	7.538 μg/g	methanol	[36]
(±)-hydramacrophyllol A	*S. judaica*	3.571 μg/g	chloroform:methanol (9:1)	[25]
	*S. tomentosa*	31.91 μg/g	methanol	[36]
(±)-hydramacrophyllol B	*S. tomentosa*	43.97 μg/g	methanol	[36]
	*S. judaica*	6.429 μg/g	chloroform:methanol (9:1)	[25]
(±)-3-(4-hydroxybenzyl)-7-hydroxyphthalide (scorzoveratrin)	*S. latifolia*	25.83 μg/g	methanol	[67]
	*S. veratrifolia*	65 μg/g	methanol	[64]
Scorzoveratrin 4′-O-β-glucoside	*S. latifolia*	8.833 μg/g	methanol	[67]
3-(4-β-glucopyranosyloxybenzyl)-7-methoxyphthalide (scorzoveratrozit)	*S. latifolia*	50 μg/g	methanol	[67]
	*S. veratrifolia*	333.333 μg/g	methanol	[64]
**Polysaccharides**				
Inulin	*S. hispanica*	226.4 μg/g	water	[60]
**Sesquiterpene lactones**				
(3*aS*,6*aR*,8*S*,9*aR*,9*bS*)-3,6,9-trimethylidene-8-[(2*R*,3*R*,4*S*,5*S*,6*R*)-3,4,5-trihydroxy-6-(hydroxymethyl)oxan-2-yl]oxy-3a,4,5,6a,7,8,9a,9b-octahydroazuleno[4,5-b]furan-2-one (glucozaluzanin C)	*S. austriaca*	N/D	acetone	[33]
(3*aS*,6*aR*,8*S*,9*aR*,9*bS*)-8-hydroxy-3,6,9-trimethylidene-3*a*,4,5,6*a*,7,8,9*a*,9*b*-octahydroazuleno[4,5-*b*]furan-2-one (zaluzanin C)	*S. austriaca*	N/D	acetone	[33]
(3*aS*,6*aR*,9*aR*,9*bS*)-3,6,9-trimethylidene-3*a*,4,5,6*a*,7,8,9*a*,9*b*-octahydroazuleno[4,5-*b*]furan-2-one (dehydrocostus lactone)	*S. austriaca*	N/D	acetone	[33]
11β,13-dihydrozaluzanin C	*S. austriaca*	N/D	acetone	[33]
14-isovaleroxyscorzoaustricin	*S. austriaca*	4.286 μg/g	acetone	[33]
14-isovaleroxyscorzoaustricin sulfate	*S. austriaca*	7.143 μg/g	acetone	[33]
4-*epi*-dihydroestafiatol	*S. austriaca*	4.286 μg/g	acetone	[33]
Biguaiascorzolide A	*S. austriaca*	5.714 μg/g	acetone	[17]
Biguaiascorzolide B	*S. austriaca*	0.857 μg/g	acetone	[17]
Diacetoxyisolippidiol	*S. austriaca*	N/D	acetone	[33]
Scorzoaustriacin	*S. austriaca*	7.143 μg/g	acetone	[33]
Scorzoaustriacin 3-*O*-β-D-glucoside	*S. austriaca*	10 μg/g	acetone	[33]
Scorzoaustriacoside	*S. austriaca*	5.714 μg/g	acetone	[33]
**Sesquiterpenoids**				
(1*R*,5*S*,6*S*,7*R*,8*S*)-8-sulfoxyguaia-4(15),10(14),11(13)-trine-6,12-olide (sulfoscorzonin A)	*S. divaricata*	0.546 μg/g	95% aqueous ethanol	[59]
(1*R*,5*S*,6*S*,7*R*,8*S*,13*S*)-8-sulfoxy-13-L-prolineyl-guaia-4(15),10(14)-dien-6a,12-olide (sulfoscorzonin C)	*S. divaricata*	7.273 μg/g	95% aqueous ethanol	[59]
(1*R*,5*S*,6*S*,7*R*,8*S*,13*S*)-8-sulfoxy-13-pyridyl-guaia-4(15),10(14)-dien-6,12-olide (sulfoscorzonin B)	*S. divaricata*	0.723 μg/g	95% aqueous ethanol	[59]
10(*Z*)-1-oxo-bisabola-2,10-dien-13-al	*S. divaricata*	1.455 μg/g	95% aqueous ethanol	[59]
1-*Oxo*-bisabola-(2,10*E*)-diene-12-al (puliglutone)	*S. hispanica*	21.786 μg/g	ethyl acetate	[20]
1-*Oxo*-bisabola-(2,10*E*)-diene-12-carboxylic acid	*S. hispanica*	14.286 μg/g	ethyl acetate	[20]
1-*Oxo*-bisabola-(2,10*E*)-diene-12-carboxylic acid methyl ester	*S. hispanica*	10.714 μg/g	ethyl acetate	[20]
1-*Oxo*-bisabola-(2,10*E*)-diene-12-ol	*S. hispanica*	3.929 μg/g	ethyl acetate	[20]
1-*Oxo*-bisabola-2-ene-12-ol (ptilostemonol)	*S. hispanica*	7.143 μg/g	ethyl acetate	[20]
2,9-Epoxycurcumen-12-al	*S. hispanica*	4.643 μg/g	ethyl acetate	[20]
Ixerisoside D	*S. hispanica*	7.021 μg/g	methanol	[32]
3β,11α-dihydroxy-4β-methyl-guaia-10(14)-en-12,6α-olide	*S. austriaca*	N/D	acetone	[16]
**Steroids**				
3β-hydroxystigmast-5-en-7-one	*S. divaricata*	1.455 μg/g	95% aqueous ethanol	[57]
3β-hydroxyl-5α,8α-epidioxyergosta-6,22-diene (5α,8α-epidioxy-(22*E*,24*R*)-ergosta-6,22-dien-3β-ol)	*S. divaricata*	3.636 μg/g	95% aqueous ethanol	[57]
5,6α-epoxy-5α-stigmastan-3β-ol	*S. divaricata*	1.273 μg/g	95% aqueous ethanol	[57]
Stigmast-3β, 5α, 6β-trihydroxy	*S. austriaca*	2.837 μg/g	acetone	[28]
Stigmast-3β, 7β-dihydroxyl-5-ene (7β-hydroxysitosterol)	*S. divaricata*	0.909 μg/g (mixture)	95% aqueous ethanol	[57]
Stigmast-3β,7α-dihydroxyl-5-ene (7α-hydroxysitosterol)	*S. divaricata*		95% aqueous ethanol	[57]
Stigmast-4-en-3-one	*S. austriaca*	2.257 μg/g	acetone	[28]
Stigmast-4-en-6β-ol-3-one (6β-hydroxystigmastan-4-en-3-one)	*S. divaricata*	1.636 μg/g	95% aqueous ethanol	[57]
Stigmasterol	*S. undulata* ssp. *deliciosa*	N/D	dichloromethane	[63]
β-Sitosterol	*S. austriaca,*	3.286 μg/g	acetone	[28]
	*S. divaricata*	6.182 μg/g	95% aqueous ethanol	[57]
	*S. latifolia*	75.714 μg/g	methanol	[3,6]
	*S. undulata* ssp. *deliciosa*	N/D	dichloromethane	[63]
	*S. veratrifolia*	N/D	methanol	[30]
β-Sitosterol 3-*O*-β-D-glucoside (β-daucosterol)	*S. austriaca*	4.314 μg/g	acetone	[28]
	*S. divaricata*	3.636 μg/g	95% aqueous ethanol	[57]
β-Stigmasterol	*S. austriaca*	1.971 μg/g	acetone	[28]
**Triterpenoids**				
(23*Z*)-Cycloart-23-en-3β,25-dihydroxy	*S. austriaca*	2.943 μg/g	acetone	[28]
(3*S*,10*R*,13*S*,14*S*,17*S*,20*S*,24*R*)-(23*E*)-3-acetoxy-25-hydroperoxy-tirucalla-7,23-diene (scorzodivaricin C)	*S. divaricata*	0.909 μg/g	95% aqueous ethanol	[59]
(3*S*,10*R*,13*S*,14*S*,17*S*,20*S*,24*R*)-3β-hydroxyl-24-hydroperoxy-24-vinyl-tirucalla-8-ene (scorzodivaricin D)	*S. divaricata*	1.091 μg/g	95% aqueous ethanol	[59]
(3*S*,5*R*,10*R*,13*S*,14*S*,17*R*,20*S*,24*R*)-3-acetoxy-24-hydroxyl-tirucalla-7,25-diene (scorzodivaricin B)	*S. divaricata*	0.909 μg/g	95% aqueous ethanol	[59]
(6*S*,7*R*)-10,11,13-trihydroxy-bisabola-2-en-1-one (scorzodivaricin A)	*S. divaricata*	0.727 μg/g	95% aqueous ethanol	[59]
23(*Z*)-3β, 25-dihydroxy-tirucalla-7,23-diene	*S. divaricata*	3.091 μg/g	95% aqueous ethanol	[59]
23(*Z*)-3β-acetoxy-25-hydroxy-tirucalla-7,23-diene	*S. divaricata*	1.273 μg/g	95% aqueous ethanol	[59]
3α-hydroxyolean-5-ene	*S. aristata*	19.036 μg/g	methanol:acetone:water (3/1/1, *v*/*v*/*v*) methanol	[56]
3β-acetoxyglutin-5(10)-en-6-oxo	*S. austriaca*	4.086 μg/g	acetone	[28]
3β-acetyl-11α,12α-oxidotaraxerol	*S. austriaca*	3.3171 μg/g	acetone	[28]
3-β-hydroxy-fern-7-en-6-one-acetate	*S. cinerea*	65 ± 1 μg/g	*n*-hexane	[27]
	*S. eriophora*	20 ± 1 μg/g	*n*-hexane	[27]
	*S. latifolia*	50 ± 1 μg/g	*n*-hexane	[27]
		7.143 μg/g	methanol	[47]
	*S. sublanata*	35 ± 1 μg/g	*n*-hexane	[27]
	*S. tomentosa*	47 ± 1 μg/g	*n*-hexane	[27]
3-β-hydroxy-fern-8-en-7-one-acetate	*S. latifolia*	N/D	methanol	[73]
9β,19-cyclolanostane- 24-en-3-oxo	*S. austriaca*	7.314 μg/g	acetone	[28]
Amyrin β-acetate	*S. undulata* ssp. *deliciosa*	10.103 μg/g	dichloromethane	[49]
D-friedours-14-en-3β-acetyl-11α,12α-epoxy	*S. austriaca*	2.229 μg/g	acetone	[28]
Fern-7-en-3-one	*S. veratrifolia*	N/D	methanol	[30]
Fern-7-en-3-β-ol (motiol)	*S. latifolia*	6.714 μg/g	methanol	[3,6]
Fern-7-en-3-β-one	*S. latifolia*	32.143 μg/g	methanol	[6]
Germanicol	*S. veratrifolia*	N/D	methanol	[30]
Germanicol acetate	*S. veratrifolia,*	N/D	methanol	[30]
Germanicone	*S. veratrifolia*	N/D	methanol	[30]
Glutinol	*S. austriaca*	4.4861 μg/g	acetone	[28]
Lupenone	*S. veratrifolia*	N/D	methanol	[30]
Lupeol	*S. acuminata*	512 ± 1 μg/g	*n*-hexane	[27]
	*S. aristata*	N/D	methanol:acetone:water (3/1/1, *v*/*v*/*v*) methanol	[56]
	*S. austriaca*	2.7431 μg/g	acetone	[28]
	*S. cana* var. *jacquiniana*	932 ± 2 μg/g	*n*-hexane	[27]
	*S. cinerea*	1073 ± 6 μg/g	*n*-hexane	[27]
	*S. eriophora*	244 ± 7 μg/g	*n*-hexane	[27]
	*S. incisa*	283 ± 2 μg/g	*n*-hexane	[27]
	*S. laciniata* ssp. *laciniata*	447 ± 2 μg/g	*n*-hexane	[27]
	*S. latifolia*	213 ± 2 μg/g	*n*-hexane	[27]
	*S. mirabilis*	224 ± 1 μg/g	*n*-hexane	[27]
	*S. mollis* ssp. *szowitsii*	282 ± 11 μg/g	*n*-hexane	[27]
	*S. parviflora*	132 ± 4 μg/g	*n*-hexane	[27]
	*S. suberosa* ssp. *suberosa*	342 ± 4 μg/g	*n*-hexane	[27]
	*S. sublanata*	415 ± 1 μg/g	*n*-hexane	[27]
	*S. tomentosa*	564 ± 2 μg/g	*n*-hexane	[27]
	*S. veratrifolia*	N/D	methanol	[30]
Lupeol acetate	*S. acuminata*	297 ± 1 μg/g	*n*-hexane	[27]
	*S. cana* var. *jacquiniana*	4273 ± 12 μg/g	*n*-hexane	[27]
	*S. cinerea*	3645 ± 8 μg/g	*n*-hexane	[27]
	*S. eriophora*	2195 ± 7 μg/g	*n*-hexane	[27]
	*S. incisa*	736 ± 10 μg/g	*n*-hexane	[27]
	*S. laciniata* ssp. *laciniata*	3212 ± 13 μg/g	*n*-hexane	[27]
	*S. latifolia*	2261 ± 94 μg/g	*n*-hexane	[27]
	*S. mirabilis*	1356 ± 2 μg/g	*n*-hexane	[27]
	*S. mollis* ssp. *szowitsii*	1244 ± 1 μg/g	*n*-hexane	[27]
	*S. parviflora*	711 ± 3 μg/g	*n*-hexane	[27]
	*S. suberosa* ssp. *suberosa*	1261 ± 5 μg/g	*n*-hexane	[27]
	*S. sublanata*	3920 ± 8 μg/g	*n*-hexane	[27]
	*S. tomentosa*	2502 ± 7 μg/g	*n*-hexane	[27]
	*S. veratrifolia*	N/D	methanol	[30]
Magnificol	*S. aristata*	N/D	methanol:acetone:water (3/1/1, *v*/*v*/*v*) methanol	[56]
Methyl oleanate	*S. undulata* ssp. *deliciosa*	N/D	dichloromethane	[63]
Methyl ursolate	*S. undulata* ssp. *deliciosa*	N/D	dichloromethane	[63]
Olean-12-en-11-one-3-acetyl	*S. cinerea*	115 ± 1 μg/g	*n*-hexane	[27]
	*S. incisa*	151 ± 1 μg/g	*n*-hexane	[27]
	*S. latifolia*	8 μg/g	methanol	[47]
		135 ± 1 μg/g	*n*-hexane	[27]
	*S. tomentosa*	187 ± 1 μg/g	*n*-hexane	[27]
Oleanolic acid	*S. divaricata*	1.818 μg/g	95% aqueous ethanol	[59]
Taraxasterol	*S. austriaca*	3.371 μg/g	acetone	[28]
	*S. veratrifolia*	N/D	methanol	[30]
Taraxasterol acetate/Taraxasteryl acetate	*S. cana* var. *jacquiniana*	719 ± 3 μg/g	*n*-hexane	[27]
	*S. cinerea*	2171 ± 6 μg/g	*n*-hexane	[27]
	*S. eriophora*	3212 ± 17 μg/g	*n*-hexane	[27]
	*S. incisa*	1191 ± 5 μg/g	*n*-hexane	[27]
	*S. laciniata* ssp. *laciniata*	276 ± 3 μg/g	*n*-hexane	[27]
	*S. latifolia,*	4201 ± 16 μg/g	*n*-hexane	[27]
	*S. mirabilis*	2099 ±4 μg/g	*n*-hexane	[27]
	*S. mollis* ssp. *szowitsii*	3791 ± 14 μg/g	*n*-hexane	[27]
	*S. parviflora*	811.96 ± 4 μg/g	*n*-hexane	[27]
	*S. suberosa* ssp. *suberosa*	2340 ± 6 μg/g	*n*-hexane	[27]
	*S. sublanata*	4981 ± 2 μg/g	*n*-hexane	[27]
	*S. tomentosa*	3168 ± 12 μg/g	*n*-hexane	[27]
	*S. veratrifolia*	N/D	methanol	[30]
Taraxasteryl myristate	*S. latifolia*	142.85 μg/g	methanol	[6]
Urs-12-en-11-one-3-acetyl	*S. latifolia*	N/D	methanol	[10]
α-Amyrin	*S. acuminata*	1646 ± 10 μg/g	*n*-hexane	[27]
	*S. cana* var. *jacquiniana*	920 ± 11 μg/g	*n*-hexane	[27]
	*S. cinerea*	3221 ± 13 μg/g	*n*-hexane	[27]
	*S. laciniata* ssp. *laciniata*	146 ± 4 μg/g	*n*-hexane	[27]
	*S. mollis* ssp. *szowitsii*	609 ± 6 μg/g	*n*-hexane	[27]
	*S. tomentosa*	969 ± 11 μg/g	*n*-hexane	[27]
α-Amyrin acetate	*S. veratrifolia*	N/D	methanol	[30]
α-Amyrin-3-acetyl	*S. austriaca*	2.857 μg/g	acetone	[28]
α-Amyrin-3-acetyl-11-oxo	*S. austriaca*	2.971 μg/g	acetone	[28]
α-Amyrinone	*S. veratrifolia*	N/D	methanol	[30]
β-Amyrin acetate	*S. veratrifolia*	N/D	methanol	[30]
β-Amyrinone	*S. veratrifolia*	N/D	methanol	[30]
β-Amyrin	*S. veratrifolia*	N/D	methanol	[30]
β-Amyrin acetate	*S. undulata* ssp. *deliciosa*	10.103 μg/g	dichloromethane	[63]
β-Amyrin-3- acetyl	*S. austriaca*	3.2 μg/g	acetone	[28]
β-Amyrin-3(3′-methylbutanoate)	*S. austriaca*	2.857 μg/g	acetone	[28]
ψ-Taraxasterol	*S. veratrifolia*	N/D	methanol	[30]
ψ-Taraxasterol acetate	*S. veratrifolia*	N/D	methanol	[30]
ψ-Taraxasteryl-3 (3′-methyl-butanonate)	*S. austriaca*	2.8 μg/g	acetone	[28]
**Tyrolobibenzyls**				
β-D-glucopyranosyl 4-[2-(4-hydroxyphenyl)ethyl]benzofuran-2-carboxylate (tyrolobibenzyl A)	*S. humilis*	701.258 μg/g	methanol	[54]
β-D-glucopyranosyl 5-hydroxy-4-[2-(4-hydroxyphenyl)ethyl]benzofuran-2-carboxylate (tyrolobibenzyl B)	*S. humilis*	829.141 μg/g	methanol	[54]
1-{3-(β-D-glucopyranosyloxy)-6-hydroxy-2-[2-(4-hydroxyphenyl)ethyl]phenyl}ethanone (tyrolobibenzyl C)	*S. humilis*	489.518 μg/g	methanol	[54]
1‴→6″-β-D-apiofuranosyl-β-D-glucopyranosyl 4-[2-(4-hydroxyphenyl)ethyl]benzofuran-2-carboxylate (tyrolobibenzyl D)	*S. humilis*	223.594 μg/g	methanol	[53]
6-*O*-β-D-glucosyl derivative of tyrolobibenzyl C (tyrolobibenzyl E)	*S. humilis*	78.189 μg/g	methanol	[55]
5-*O*-glucosyl derivative of tyrolobibenzyl B (tyrolobibenzyl F)	*S. humilis*	6.859 μg/g	methanol	[55]
**Other compounds**				
Verbascoside (acteoside)	*S. undulata* ssp. *deliciosa*	72.165 μg/g	dichloromethane	[49,63]
Methyl-β-D-fructofuranoside	*S. divaricata*	3.273 μg/g	95% aqueous ethanol	[57]
1-monolinolein (glycerol 1–9′,12′-octadecadienoate)	*S. divaricata*	2.364 μg/g	95% aqueous ethanol	[57]
2-[(*E*)-2-(4-hydroxyphenyl)ethenyl]-6-methoxybenzoic acid (scorzoerzincanin)	*S. tomentosa*	105.528 μg/g	methanol	[36]

Compound concentration was taken directly from literature or it was calculated, dividing the mass of the isolated compound by the mass of plant material used for extraction; N/D—no data was available in the literature.

**Table 3:** Compounds isolated from *Scorzonera* whole plants.

Compounds	*Scorzonera* Species	Concentration	Type of Solvent	References
**Dihydroisocoumarins and dihydroisocoumarin derivatives**				
6,8-dihydroxy-3-(4methoxyphenyl)isochroman-1-one (scorzocreticin)	*S. cretica*	13.334 μg/g	methanol	[37]
8-O-[α-L-rhamnopyranosyl-(1→6)-β-D-glucopyranosyl]scorzocreticin (scorzocreticoside II)	*S. cretica*	48.2 μg/g	methanol	[37]
8-*O*-β-D-glucopyranosylscorzocreticin (scorzocreticoside I)	*S. cretica*	23.334 μg/g	methanol	[37]
**Flavonoid aglycones**				
Apigenin	*S. undulata* ssp. *alexandrina*	9 μg/g	petroleum ether	[23]
**Steroids**				
3-*O*-β-D-glucopyranosylsitosterol	*S. cretica*	N/D	methanol	[37]
3-O-(6-O-acetyl-β-D-glucopyranosyl)β-sitosterol	*S. undulata* ssp. *alexandrina*	6 μg/g	petroleum ether	[23]
Daucosterol	*S. undulata* ssp. *alexandrina*	12 μg/g	petroleum ether	[23]
**Triterpenoids**				[37]
24-methylenecycloartanol	*S. undulata* ssp. *alexandrina*	4 μg/g	petroleum ether	[23]
Germanicol	*S. cretica*	N/D	dichloromethane	[37]
Germanicol acetate	*S. cretica*	N/D	dichloromethane	[37]
Germanicone	*S. cretica*	N/D	dichloromethane	[37]
Lupenone	*S. cretica*	N/D	dichloromethane	[37]
Lupeol	*S. cretica*	N/D	dichloromethane	[37]
	*S. undulata* ssp. *alexandrina*	5 μg/g	petroleum ether	[23]
Lupeol acetate	*S. cretica*	N/D	dichloromethane	[37]
Oleanol	*S. cretica*	N/D	dichloromethane	[37]
Oleanol acetate	*S. cretica*	N/D	dichloromethane	[37]
Taraxasterol	*S. cretica*	N/D	dichloromethane	[37]
Taraxasterol acetate/Taraxasteryl acetate	*S. cretica*	N/D	dichloromethane	[37]

Compound concentration was taken directly from literature or it was calculated, dividing the mass of the isolated compound by the mass of plant material used for extraction; N/D—no data was available in the literature.

## Data Availability

Not applicable.

## Abbreviations

ALT	alanine transaminase
AST	aspartate transaminase
BCB	beta-carotene bleaching
CCl_4_	carbon tetrachloride
CHCl_3_	chloroform
COX-1	cyclooxygenase 1
COX-2	cyclooxygenase 2
CUPRAC	cupric reducing antioxidant capacity
FRAP	ferric reducing antioxidant activity
H_2_O_2_	hydrogen peroxide
H_2_SO_4_	sulfuric acid
HNO_3_	nitric acid
Hyp	hydroxyproline
IC_50_	half-maximal inhibitory concentration
IL-1β	interleukin 1β
MBC	minimal bactericidal concentration
MIC	minimal inhibitory concentration
NF-κB	nuclear factor kappa B
OSI	oxidative stress index
RACI	relative antioxidant capacity index
SC_50_	half-maximal scavenging concentration
TAS	total antioxidant status
TEAC	Trolox equivalent antioxidant capacity
TNF-α	tumor necrosis factor α
TOS	total oxidant status
TPC	total phenolic content
